# Differences in the 3’ intergenic region and the V2 protein of two sequence variants of tomato curly stunt virus play an important role in disease pathology in *Nicotiana benthamiana*

**DOI:** 10.1371/journal.pone.0286149

**Published:** 2023-05-23

**Authors:** Alexander M. Zwolinski, Alison Brigden, Marie E. C. Rey

**Affiliations:** School of Molecular and Cell Biology, University of the Witwatersrand, Johannesburg, South Africa; ICAR-Central Plantation Crops Research Institute, INDIA

## Abstract

Tomato production in South Africa is threatened by the emergence of tomato curly stunt virus (ToCSV), a monopartite *Begomovirus* transmitted by the whitefly vector *Bemisia tabaci* (Genn.). We investigated the role of sequence differences present in the 3’ intergenic region (IR) and the V2 coding region on the differing infectivity of ToCSV sequence variant isolates V30 and V22 in the model host *Nicotiana benthamiana*. Using virus mutant chimeras, we determined that the development of the upward leaf roll symptom phenotype is mediated by sequence differences present in the 3’ IR containing the TATA-associated composite element. Sequence differences present in the V2 coding region are responsible for modulating disease severity and symptom recovery in V22-infected plants. Serine substitution of V22 V2 Val27 resulted in a significant increase in disease severity with reduced recovery, the first study to demonstrate the importance of this V2 residue in disease development. Two putative ORFs, C5 and C6, were identified using in silico analysis and detection of an RNA transcript spanning their coding region suggests that these ORFs may be transcribed during infection. Additional virus-derived RNA transcripts spanning multiple ORFs and crossing the boundaries of recognised polycistronic transcripts, as well as the origin of replication within the IR, were detected in ToCSV-infected plants providing evidence of bidirectional readthrough transcription. From our results, we conclude that the diverse responses of the model host to ToCSV infection is influenced by select sequence differences and our findings provide several avenues for further investigation into the mechanisms behind these responses to infection.

## Introduction

Tomato (*Solanum lycopersicum* L.) is a versatile agricultural food crop belonging to the dicotyledonous plant family *Solanaceae* and is grown exclusively for its fruit which is considered a culinary vegetable [1]. It ranks second in economic importance as a solanaceous crop after potato [2]. The nutritious tomato fruit is rich in minerals, vitamins, and carotenoids [3]. In South Africa (SA), the commercial industry is composed of nearly 700 producers and as of 2011, supplied 95% of the total annual fruit output and employed over 22,000 people [4]. As tomato is largely harvested by hand, the industry provides employment opportunities for low-skilled agricultural labourers [5].

Tomato is highly susceptible to infection by several viruses transmitted by the whitefly *Bemisia tabaci* (Genn.). A recent survey of tomato-growing regions in SA revealed that infestations of this polyphagous insect pest are widespread and particularly severe in the Limpopo province which is a major tomato producing region [6]. Tomato yellow leaf curl viru*s* (TYLCV) and an ever-expanding group of related whitefly-transmitted viruses having circular ssDNA genomes belonging to the genus *Begomovirus* (family *Geminiviridae*) continue to cause devastating losses to the tomato crop worldwide [7–9]. Monopartite begomoviruses contain a single ssDNA genomic component ~2.6 kb in size whilst bipartite begomovirus genomes are composed of two ssDNA components (DNA-A and DNA-B) each ~2.6 kb in size [10]. *Tomato curly stunt virus* is a distinct tomato-infecting monopartite begomovirus species closely related to a group of solanaceous plant host-infecting begomoviruses from sub-Saharan Africa and Southwest Indian Ocean islands [11–13]. Tomato curly stunt virus (ToCSV) was first detected in the Onderberg region of Mpumalanga province but has since spread to all tomato growing regions of SA and can cause up to 100% yield losses in susceptible cultivars [14–16]. ToCSV induces similar symptoms to TYLCV in tomato, including yellowing of the young upper leaves, curling and cupping of leaflet margins, severe stunting and dwarfing of overall plant growth as well as decreased fruit set [11, 14]. A study of several tomato cultivars which are known to show resistance to TYLCV were found to also show some levels of resistance to ToCSV [15].

The genome of ToCSV contains six canonical ORFs found in other OW monopartite begomoviruses [10, 14, 17]. Two are located on the virion-sense (vs) strand: V1 encoding the coat protein (CP) and V2 encoding the pre-coat protein (V2). Four are located on the complementary-sense (cs) strand: C1 encoding the replication-associated protein (Rep), C2 encoding the transcriptional activator (TrAP), C3 encoding the replication enhancer (REn), and C4 encoding the C4 protein [10]. The identification of additional putative ORFs (C5/AC5 and C6) in begomoviruses has been reported previously [18–20]. The intergenic region (IR) present between the C1 and V2 ORFs contains a stem-loop element containing a conserved nonanucleotide sequence (5’-TAATATTAC-3’) at the origin of replication (Ori) [17, 21]. The IR contains several promoters and *cis*-acting elements on either side of the stem-loop that are involved in regulating the expression of vs and cs ORFs [22–24]. The IR also contains repeat sequence (iteron) motifs present in the 5’ IR situated in the vicinity of the TATA box core promoter sequence which act as binding sites for Rep which binds viral DNA to initiate cleavage at the Ori of the vs strand [25, 26].

The 3’ end of the IR terminates at the start codon for the V2/AV2 ORF which is present in OW begomoviruses only [17]. The V2/AV2 protein is known to play a key role in virus pathogenicity and exhibits a multifunctional nature that contributes to virus-host interactions. Early studies of monopartite geminiviruses provided evidence that an intact V2 protein is required for successful systemic infection, implicating it in facilitating movement within the host [27–30]. Potato virus X (PVX)-vector based expression of several geminivirus V2/AV2 ORFs in *Nicotiana benthamiana* has demonstrated that not only is V2/AV2 able to induce severe virus-like symptoms such as leaf curling and chlorosis but has also been implicated to function as an avirulence factor that promotes the development of a hypersensitive response [31–34]. Several geminivirus V2/AV2 proteins have been demonstrated to function as pathogenicity determinants necessary for inducing severe disease [31, 32, 34–39]. In addition, V2/AV2 proteins have been reported to act as potent suppressors of transcriptional and post-transcriptional gene silencing (TGS and PTGS) by several modes of action. These include competitive binding with important host proteins involved in antiviral defence, dsRNA binding to prevent siRNA biogenesis, as well as siRNA sequestration activity [38, 40–46].

A survey of tomato-growing regions in South Africa resulted in the discovery of two ToSCV sequence variant groups (ToCSV-I and ToCSV-II), differentiated by the presence of a recombinant fragment in the genomic region spanning part of the 3’ IR and the first half of the V2 ORF in ToCSV-II isolates [16]. In the present study, we investigated the genomic coding capacity and in vivo infectivity of single representative isolate genome sequences from both variant groups. Through generation of virus mutant chimeras, we identified key sequence differences present in the IR and V2 which modulate symptom phenotype, viral titre, and disease recovery in infected *N*. *benthamiana*.

## Materials and methods

### ToCSV infectious clone constructs

Agroinfectious clone constructs of the wild type ToCSV-I isolate ToCSV-[ZA:Mks30:08] (GenBank Accession No. OK813888.1) and ToCSV-II isolate ToCSV-[ZA:Mks22:07] (GenBank Accession No. OK813889.1) in pCAMBIA2300 were constructed previously [16]. The ToCSV-I and ToCSV-II isolates herein referred to as “V30” and “V22” respectively, shared 96% nt sequence identity. V30 was shown to induce severe disease in tomato ‘Rooikhaki’, whilst V22 caused milder disease symptoms in this tomato cultivar [16]. Four partial IR sequence swap mutants and two full V2 ORF sequence swap mutants were designed and are described in S1 Table. The V30ΔIR-s, V22ΔIR-s, V22ΔIR-sr, V30ΔV2-s, and V22ΔV2-s mutants were synthesised by Genscript Biotech (Piscataway, USA). The V30ΔIR-sr mutant was synthesised by Inqaba Biotech (Pretoria, SA). The identities of the mutated regions of the swap mutant clones were confirmed using PCR with Sanger sequencing. Details of sequencing primers used are indicated in S2 Table.

### Phylogenetic and amino acid sequence analyses

Full genome nt sequences of V30 and V22 were aligned with selected African tomato-infecting monopartite begomoviruses to generate a pairwise nt sequence identity matrix (MUSCLE alignment) using SDT v 1.2 software [47]. A multiple nt sequence alignment of the conserved sequence elements identified in the 3’ IR spanning the TATA box to the V2 start codon of the two ToCSV variant sequences as well as selected African begomoviruses sharing ≥78% nt sequence identity with ToCSV was manually constructed. The full genome sequences of V30 and V22 were submitted to the ORF finder server (https://www.ncbi.nlm.nih.gov/orffinder/) to identify ORFs predicted to encode proteins ≥60 aa in length when following standard code. Translated protein sequences were subjected to comparison with other geminivirus protein sequences using BLAST [48]. Pairwise V30 and V22 protein aa sequence alignments were generated using PRALINE multiple sequence alignment server (https://www.ibi.vu.nl/programs/pralinewww/). Details of all viral isolates used in analyses are shown in S3 Table.

### Site-directed mutagenesis

The V22 infectious clone was used as a template to substitute V22 V2 Val27 by a serine (V22ΔV2-V27S) and V22 V2 Thr58 by a serine (V22ΔV2-T58S). Site-directed mutagenesis was done using Phusion™ High-Fidelity DNA Polymerase (Thermo Fisher Scientific). Partially overlapping mutagenesis primer pairs were manually designed following published recommendations with amendments [49–51]. Target mutations were included in both primers, Tm differences for all primer pairs did not exceed 2°C, and primer GC content ranged from 38 to 50%. The OligoAnalyzer™ tool (https://www.idtdna.com/oligoanalyzer) was used to determine predicted hairpin, homo- and heterodimer ΔG values. The accepted cut-offs for predicted ΔG values were -2.0 kcal/mol for hairpins, and -9.0 kcal/mol for dimers. Primer sequence lengths were adjusted until acceptable ΔG values were obtained whilst following strict published mutagenesis primer requirements. Mutagenesis primers were synthesised, and RP-cartridge purified by Inqaba Biotech (Pretoria, SA). Mutagenesis PCR was set up as per manufacturer’s instructions using 4 ng template DNA with 1% DMSO being included (except in ΔV2T58S_FP/RP PCR reaction) to inhibit secondary structure formation. A three-step cycling protocol was followed using 20 cycles and an extension time of 520 s (45 s/kb). The annealing temperatures used were 65°C for ΔV2V27S_FP/RP PCR, and 69°C for ΔV2T58S_FP/RP. Upon completion, 5 μL PCR product samples were resolved on 0.7% agarose to verify the presence of the target products at ~12 kbp. Restriction digestion using FastDigest DpnI (Thermo Fisher Scientific) was done by adding 1 μL enzyme directly to each of the remaining PCR product samples and incubating at 37°C for 15 h followed by heat inactivation. Preparation and transformation of ultracompetent *Escherichia coli* XL10-Gold cells was done as per Green and Sambrook [52] with the following modifications: 2 uL of DpnI-digested sample was added directly to 100 μL cells and β-mercaptoethanol (24 mM final concentration) was included to enhance transformation efficiency. Transformed cells were plated on Super Optimal Broth (SOB) agar containing 100 μg/mL kanamycin and incubated at 37°C for 24 h. Three to five colonies were selected and sub-cultured, and plasmid DNA was extracted and quantified. All mutant clones generated in this study were confirmed using PCR and Sanger sequencing. ORF finder was used to confirm that no inadvertent mutations had been introduced into the overlapping ORFs. Details of mutagenesis and sequencing primers used are indicated in S2 Table.

### Plant material and growth conditions

Wild type *N*. *benthamiana* was seeded in Jiffy® 7C cocopeat pellets in a controlled environment facility with a 16 h light/8 h dark photoperiod, relative humidity ~50% and an ambient temperature of 26 ± 2°C. Germination was induced under clingwrap and 30-day-old seedlings were acclimatised over a period of 4 to 7 days prior to inoculation at the 5 to 6-leaf stage. Plants were transferred in Jiffy® pellets to pre-wet cocopeat four days post inoculation (dpi). Supplementation with 20 mL per plant with Kelpak seaweed extract was done at 4 dpi and Seagro organic fertiliser at 8, 16, 26, and 36 dpi.

### Inoculum preparation and plant agroinoculation

Competent *Agrobacterium tumefaciens* C58C1 cells were prepared and subsequently transformed with purified infectious clone construct plasmid DNA in accordance with Höfgen and Willmitzer [53]. Transformed cells were plated on LB agar containing 100 μg/mL rifampicin and 100 μg/mL kanamycin and incubated at 28°C for 48 h. Positive transformants were identified and sub-cultured in LB broth with antibiotics until OD_600_ ~1.0 was obtained. To prepare inoculum, cultures were centrifuged and resuspended in chilled sterile 0.1 M MgCl_2_ containing 100 μM acetosyringone to OD_600_ 0.8. Cell suspensions were incubated in the dark at ambient temperature for up to 3 h with occasional gentle shaking. Plants were inoculated with 100 μL prepared inoculum using a 1 mL needleless syringe, with gentle infiltration of the underside of two expanded leaves below the meristem. Five plants were inoculated per treatment. Plants inoculated with *A*. *tumefaciens* C58C1 harbouring empty pCAMBIA2300 vector served as the mock-inoculated controls. Infection was confirmed using PCR with primers targeting a conserved region of the V2 ORF and subsequent Sanger sequencing. DNA extracted at 28 dpi from plants infected with V2 site-directed mutants was used as a template for PCR with Sanger sequencing to confirm that mutation reversion to wild type sequence had not occurred.

Preliminary infections of *N*. *benthamiana* with wild type ToCSV infectious clone constructs were conducted to develop a novel symptom severity scoring system. Two symptoms of interest were identified: upward leaf roll (ULR), and swollen veins (SV). The ULR disease symptom initially presented as upward turning of the edges of the emerging leaf, gradually progressing to upward rolling of the leaf edges as the leaf expanded. Increased severity of ULR was associated with leaf roll being present along the entire leaf edge or broad leaf roll that resulted in the upward rolling of the entire leaf surface. For scoring of ULR symptoms, the leaf edges were demarcated into seven zones (S1 Fig). The SV phenotype manifested as abnormal swelling of the leaf veins resulting in abaxial vein bulging and adaxial vein depression. This symptom was especially apparent when viewed through a light source as an enhanced visual contrast of the vein network was observed. Severe SV symptoms presented as a dense network of affected leaf veins often associated with rugosity and leaf distortion. Milder SV presented as sparsely distributed SV or slight SV present near the leaf edges without associated rugosity. The visual scoring systems for ULR and SV are described in S4 and S5 Tables, respectively. All inoculated plants were observed daily for symptom development. Symptom scoring was done at four-day intervals starting at 12 dpi; three expanded leaves below the meristem were scored for each plant. Plant trials were terminated after 36 dpi. Symptom scoring was done for three biological and 15 technical replicates. Student’s t-tests were conducted using IBM SPSS Statistics v 28.0 software package to determine statistically significant differences between corresponding symptom scores in different treatments.

### Nucleic acid extraction

For DNA extraction, two leaf discs (d = 12.7 mm) were collected from expanded leaves below the meristem at 12, 20, 28 and 36 dpi. Leaf tissue was flash frozen in liquid nitrogen and homogenised with 3 mm tungsten carbide beads (Qiagen) using the Qiagen TissueLyser II system (Qiagen) at 20 Hz for 1 min twice. CTAB extraction was done in accordance with Doyle [54] with the following modifications: an additional chloroform-isoamyl alcohol extraction step, two ethanol wash steps (95% first wash, 75% second wash), and incubation of samples at -80°C for 30 min after the addition of isopropanol.

For total RNA extraction, ~100 mg of homogenized leaf material collected at 20 dpi was flash frozen in liquid nitrogen and homogenized with DEPC-treated 3 mm tungsten carbide beads (Qiagen) using the Qiagen TissueLyser II system (Qiagen) at 20 Hz for 1 min twice. RNA was extracted using TRIzol^TM^ reagent (Thermo Fisher Scientific) as per the manufacturer’s protocol with an additional chloroform separation step. All nucleic acids were quantified using the NanoDrop^TM^ One spectrophotometer (Thermo Fisher Scientific) and purity was also assessed using gel electrophoresis. Extracted DNA and RNA was stored at -80°C for downstream analysis.

### Quantitative PCR (qPCR)

To determine viral load in inoculated plants at specified time points, real-time quantitative PCR (qPCR) was done using the Maxima SYBR Green/ROX qPCR Master Mix (Thermo Fisher Scientific) as per manufacturer’s protocol with the CFX96 Touch Real-Time PCR Detection System (Bio-Rad Laboratories). Viral load was determined relative to the internal control *glyceraldehyde 3-phosphate dehydrogenase* (*GAPDH*) [55]. The degenerate primer pair used to target viral V2 ORF was designed using Primer3 with appropriate qPCR primer parameters selected [56]. The primer sequences used are shown in S2 Table. Primer specificity was confirmed using standard PCR. Primer amplification efficiencies were determined through construction of standard curves (S2 Fig) using CFX Maestro Software v 1.0 (Bio-Rad Laboratories). For qPCR experiments, three biological and nine technical replicates were used. Log viral fold change was calculated using the delta Ct method (Log ΔCt = 2^-ΔCt^) adapted from Livak and Schmittgen [57]. Student’s t-tests were conducted using IBM SPSS Statistics v 28.0 software package to determine statistically significant differences between viral load in different treatments.

### Reverse transcription PCR (RT-PCR)

To investigate the presence of virus-derived RNA transcripts in infected *N*. *benthamiana*, strand-specific primers for cDNA synthesis were designed using Primer3 [56]. Primer sequences were evaluated for the presence of secondary structures using the Oligoanalyzer^TM^ Tool (https://www.idtdna.com/oligoanalyzer). For details of primers used refer to S2 Table. First strand cDNA was synthesized using the RevertAid™ First Strand cDNA Synthesis Kit (Thermo Fisher Scientific) following the manufacturer’s protocol using gene-specific primers with inclusion of all prescribed negative controls and mock-inoculated control. All cDNA was stored at -80°C until subsequent PCR analysis was done using DreamTaq™ PCR Master Mix (Thermo Fisher Scientific) in accordance with the manufacturer’s instructions. All RT-PCR products were resolved on 2% agarose. RT-PCR was repeated three times.

## Results

### Investigation in silico of two ToCSV sequence variant isolate genomes

**Fig 1:**
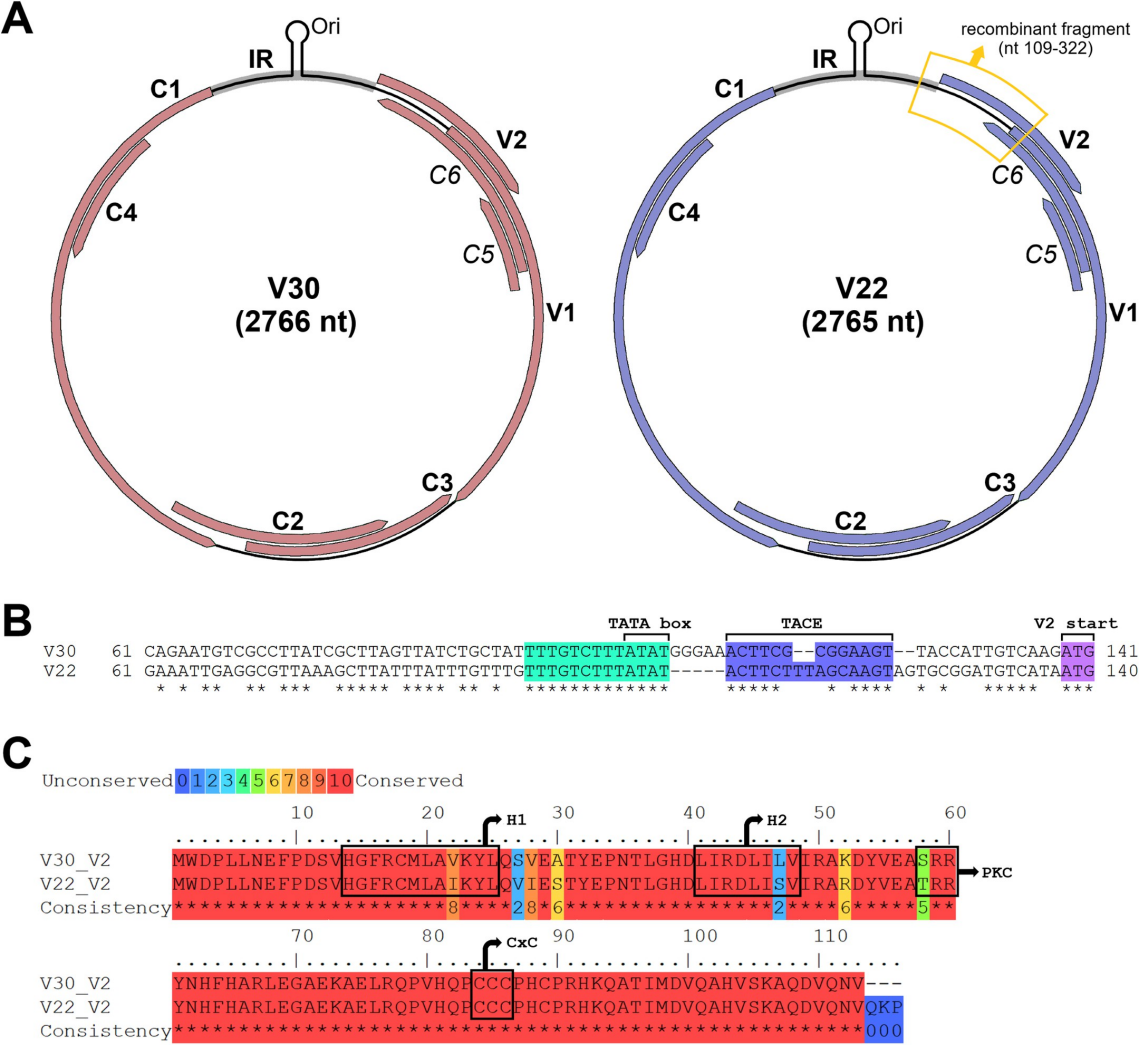
Comparison of ToCSV sequence variant isolates V30 and V22. (A) Genome organisation of V30 (left) and V22 (right). Accepted virion-sense and complementary-sense ORFs indicated with bold labels, putative C5 and C6 ORFs indicated with italicised labels. Intergenic region (IR) highlighted in grey with stem-loop element containing origin of replication (Ori) shown. Recombinant fragment present in V22 spanning part of the 3’ IR and the first half of the V2 ORF boxed in yellow. (B) Pairwise partial 3’ IR nt sequence alignment for V30 and V22 done using MUSCLE alignment program with manual adjustments. Conserved region containing TATA box in green. TATA-associated composite element (TACE) in blue. V2 start codon in magenta. (C) Pairwise V2 protein aa sequence alignment generated using PRALINE with colour key indicating aa conservation. Hydrophobic domains (H1 and H2), putative protein kinase C (PKC) phosphorylation motif, and CxC motif boxed in black. Hyphens indicate alignment-generated gaps. Asterisks indicate sequence conservation.

Both V30 and V22 genome sequences were predicted to exhibit the genome organisation typical of other OW monopartite begomoviruses [10]. Two additional putative ORFs present on the cs strand, herein termed C5 and C6, were also identified. The six canonical and two putative ORFs were each mapped to the virus genomes (Fig 1A) and the nt positions of start/stop codons and predicted protein lengths were obtained for both V30 and V22 ORFs (S6 Table). The C5 start codon was situated upstream of the C6 start codon. Sequence analysis revealed that C5 was in frame with V1, and C6 was in frame with V2. The C5 proteins of both V30 and V22 were 60 aa in length (S3A Fig), and a search of the Conserved Domain Database [58, 59] determined that both contained the ‘Gemini AC5-2 domain’ (pfam08464) previously described by Li et al. [19]. Pairwise V30 and V22 C6 protein aa sequence alignment revealed significant sequence differences in the C-terminal region (S3B Fig). The V30 C6 protein (143 aa) had an extended C-terminus (35 aa) that was absent from the V22 C6 protein (108 aa).

Although both V30 and V22 shared 96% nt sequence identity, several nt sequence differences were present. Pairwise sequence alignment of V30 and V22 partial 3’ IR sequences (Fig 1B) revealed the presence of two short regions having sequence differences separated by a conserved TATA box-containing region (nt 95 to 107) upstream of the V2 start codon. The recombinant fragment present in V22 and other ToCSV-II isolates [16] included the 3’ IR region to the right of the TATA box. This region contained the TATA-associated composite element (TACE), a novel *cis*-acting element described by Cantú-Iris et al. [60]. A multiple nt sequence alignment of the short TACE-containing region of ToCSV and related African tomato-infecting monopartite begomoviruses was done to compare the sequence and arrangement of the TACE (S4 Fig). This regulatory element was identified in all begomovirus isolates except for tomato leaf curl Toliara virus (AM701768). The central spacer region of the TACE between the conserved left (ACTT) and right (AAGT) arms was highly variable. Multiple sequence comparison uncovered two features of the TACE found only in the ToCSV exemplar isolate (AF261885) and V30. The first feature was an additional spacer sequence (GGGAA) between the TATA box and the conserved left arm sequence of the TACE. The second feature was a reduced TACE central spacer region containing five nt instead of the consensus seven nt present in the TACE of V22 and other African tomato-infecting begomoviruses (S4 Fig).

The V22 recombinant fragment encompassed the V2 ORF region coding for the N-terminal 62 aa. This region of the V30 and V22 V2 proteins contained seven aa sequence differences (Fig 1C). Two hydrophobic domains (H1 and H2) and the putative protein kinase C (PKC) phosphorylation motif which have been characterised previously [31, 34, 35] were mapped to this variable N-terminal region (Fig 1C). The C-terminal half of V2 contained a CxC motif [31, 33] conserved between the two V2 proteins whilst the V2 of V22 also contained three additional C-terminal residues (Gln-Lys-Pro) absent in V30 V2 (Fig 1C).

### Sequence swapping of the 3’ IR results in a change in symptom phenotype

To identify the role of 3’ IR sequence differences in disease symptom expression, we inoculated *N*. *benthamiana* with IR sequence swap mutant chimeras (S1 Table) and compared with wild type virus-infected plants. At ~12 dpi, virus-like symptoms began to appear in the emerging leaves of plants agroinoculated with virus infectious clones. Leaf epinasty, rugosity, mild chlorosis, and leaf curling with associated disruption in flower development was observed.

**Fig 2:**
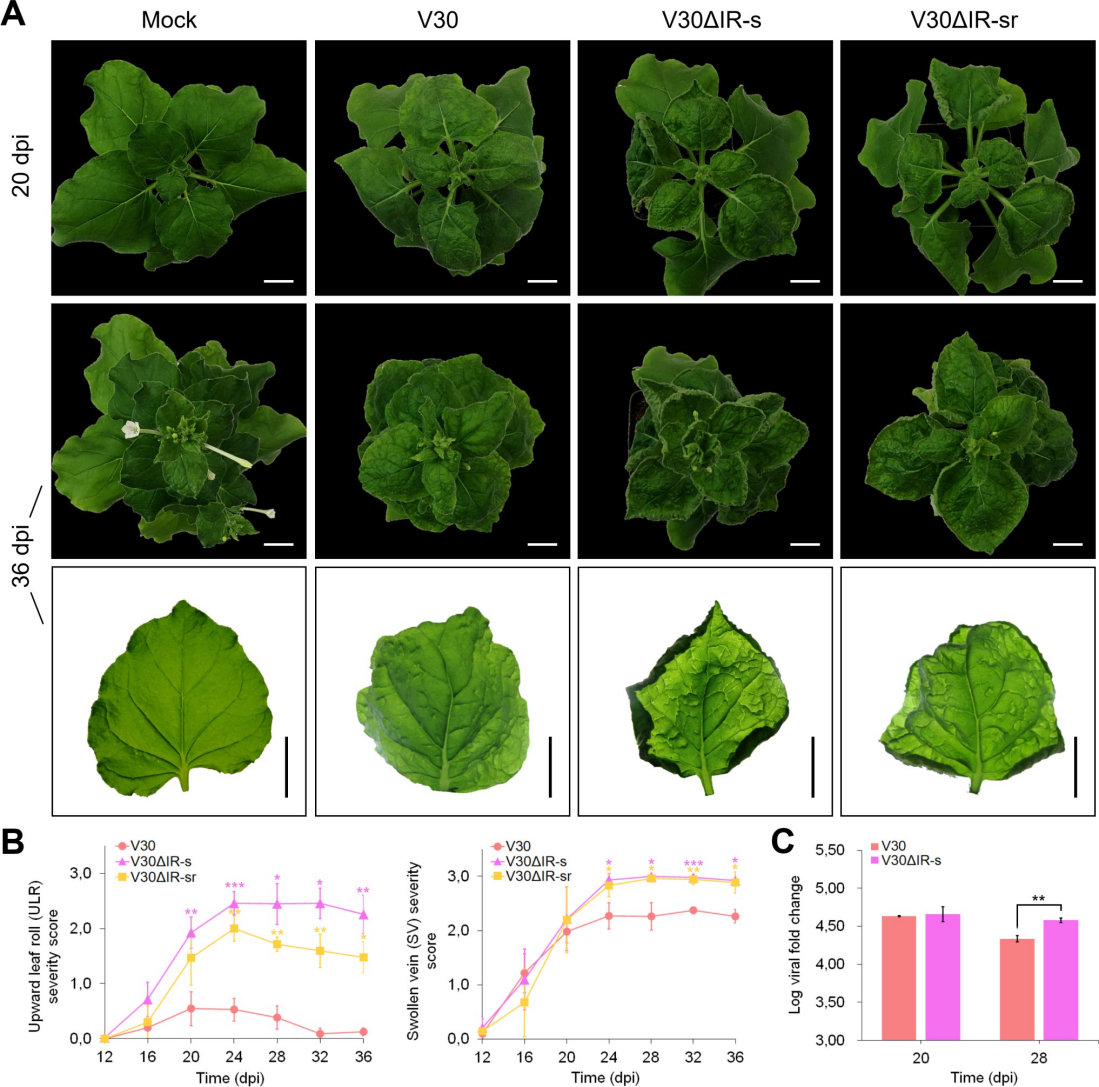
Single infection of *Nicotiana benthamiana* with V30 intergenic region (IR)-swap mutant infectious clones. (A) Symptoms in plants inoculated with V30, V30ΔIR-s, and V30ΔIR-sr at 20 dpi (top row) and 36 dpi (middle and bottom rows). Bar = 2 cm. Mock inoculated with *Agrobacterium tumefaciens* C58C1 carrying empty pCAMBIA2300. (B) Corresponding upward leaf roll (ULR) and swollen vein (SV) severity scores at four-day intervals from 12 to 36 dpi. (C) Log viral fold change at 20 and 28 dpi. Bars represent mean ± SD. Student’s t-test levels of significance: *, *p* < 0.05; **, *p* < 0.01; ***, *p* < 0.001.

In V30-infected plants, upward leaf roll (ULR) symptoms were mild to almost absent over the entire monitoring period (Fig 2A and 2B). Plants infected with V30 also displayed moderate to severe swollen vein (SV) symptoms by 20 dpi with no reduction in symptom severity in the emerging leaves by 36 dpi (Fig 2A and 2B). Mock-inoculated plants developed normally without disease symptoms and were observed to flower by 36 dpi (Fig 2A). Agroinoculation of *N*. *benthamiana* with V30ΔIR-s (nt 61–138 swapped) and V30ΔIR-sr (nt 108–138 swapped) resulted in significant changes to the wild type V30 symptom phenotype. Severe and persistent ULR symptoms were observed to develop in plants inoculated with these two IR-swap mutants (Fig 2A and 2B). The severity of SV symptoms was also significantly increased between 24 and 36 dpi when compared to V30-infected plants. A dense network of SV was associated with severe leaf rugosity. Both ULR and SV persisted without recovery in IR-swap mutant-infected plants by 36 dpi (Fig 2A and 2B). ULR symptom severity was significantly higher in plants infected with V30ΔIR-s than V30ΔIR-sr at 28 and 36 dpi although both mutants induced identical SV symptom severity. Viral load relative to *GAPDH* was determined in V30 and V30ΔIR-s-infected plants using qPCR. A significantly higher viral titre was recorded at 28 dpi in plants infected with V30ΔIR-s than in wild-type V30-infected plants (*p* = 0.003) (Fig 2C).

**Fig 3:**
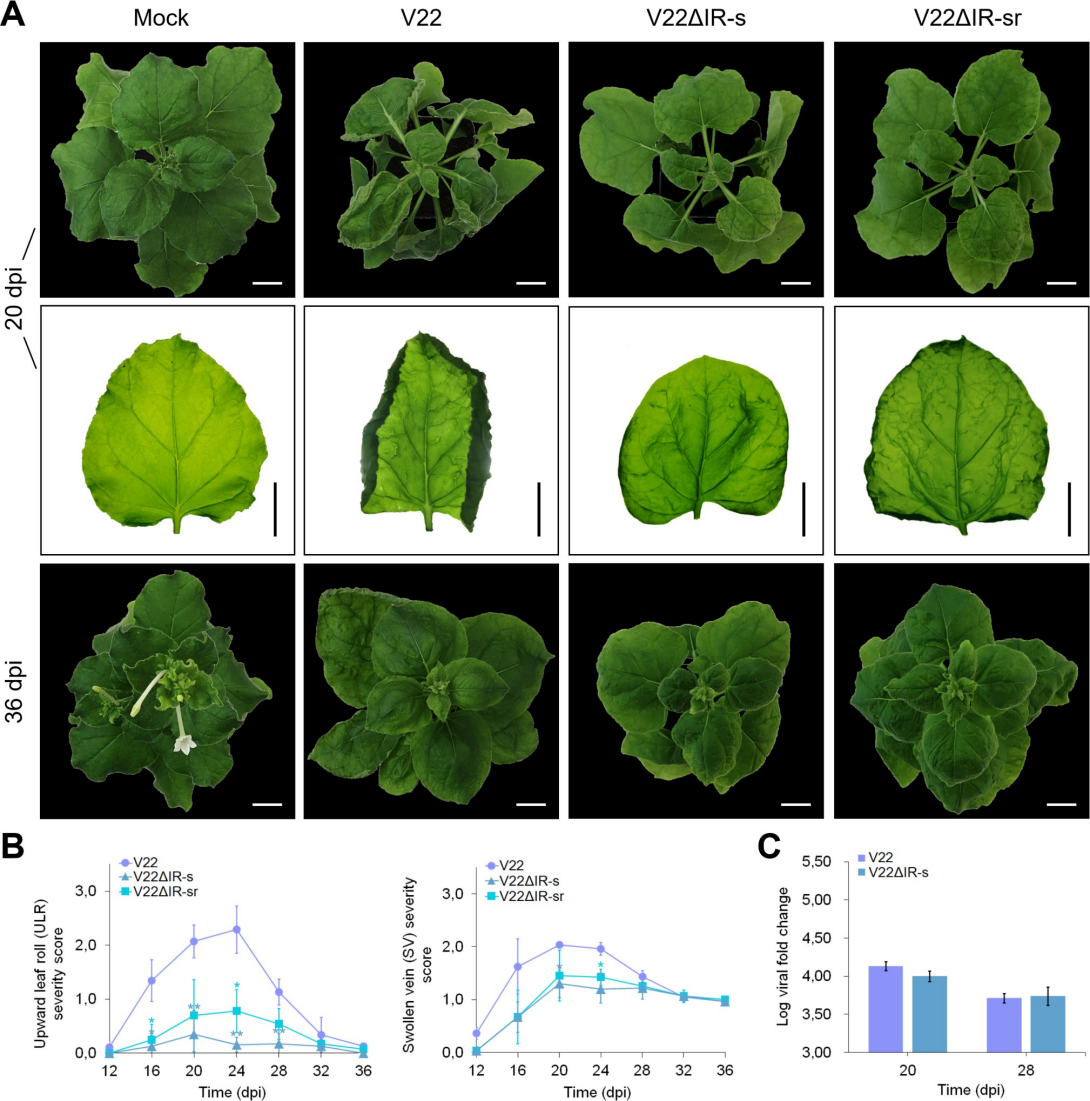
Single infection of *Nicotiana benthamiana* with V22 intergenic region (IR)-swap mutant infectious clones. (A) Symptoms in plants inoculated with V22, V22ΔIR-s, and V22ΔIR-sr at 20 dpi (top and middle rows) and 36 dpi (bottom row). Bar = 2 cm. Mock inoculated with *Agrobacterium tumefaciens* C58C1 carrying empty pCAMBIA2300. (B) Corresponding upward leaf roll (ULR) and swollen vein (SV) severity scores at four-day intervals from 12 to 36 dpi. (C) Log viral fold change at 20 and 28 dpi. Bars represent mean ± SD. Student’s t-test levels of significance: *, *p* < 0.05; **, *p* < 0.01.

Plants infected with V22 developed moderate to severe ULR and SV symptoms peaking in severity from 20 to 24 dpi. A symptom recovery phenotype was observed as a significant reduction in SV and ULR severity in V22-infected plants between 24 and 36 dpi, with ULR expression being reduced to near zero whilst SV persisted at mild severity (Fig 3A and 3B). The expression of ULR symptoms was almost completely abolished in plants infected with V22ΔIR-s (nt 61–137 swapped) (Fig 3A and 3B). ULR severity was also reduced to a lesser extent in plants agroinoculated with V22ΔIR-sr (nt 108–137 swapped) (Fig 3A and 3B). A reduction in the peak mean SV severity was also recorded in plants infected with V22ΔIR-s and V22ΔIR-sr, whilst observed disease severity was identical to that of V22-infected plants by 32 dpi. No significant difference was observed in ULR and SV symptom severity between plants infected with V22ΔIR-s and V22ΔIR-sr. Viral load in plants infected with V22ΔIR-s was found not to be significantly different from viral load measured in V22-infected plants (Fig 3C).

### Sequence swapping and mutagenesis targeting the V2 coding region results in changes to disease severity

**Fig 4:**
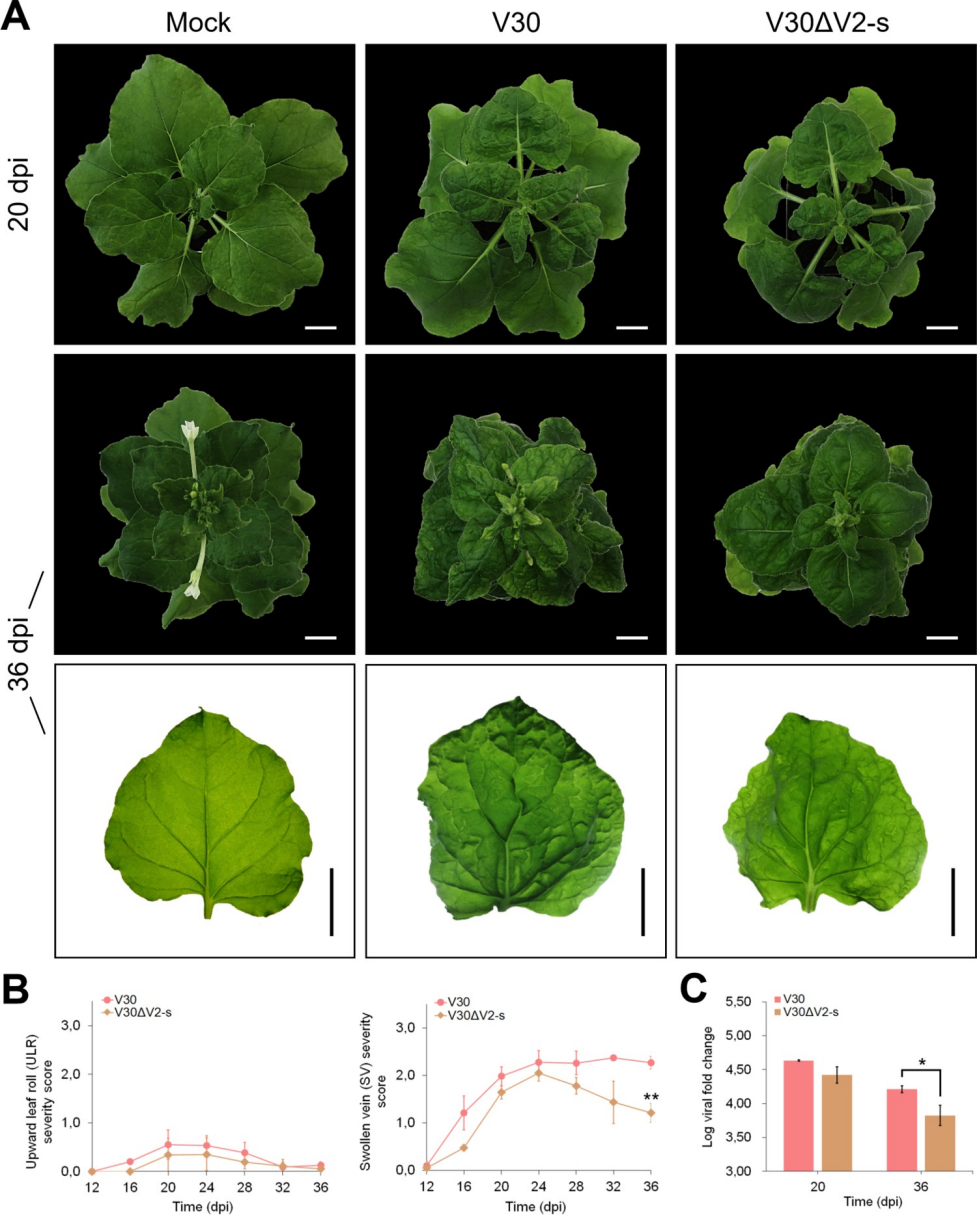
Single infection of *Nicotiana benthamiana* with V30 V2-swap mutant infectious clone. (A) Symptoms in plants inoculated with V30, and V30ΔV2-s at 20 dpi (top row) and 36 dpi (middle and bottom rows). Bar = 2 cm. Mock inoculated with *Agrobacterium tumefaciens* C58C1 carrying empty pCAMBIA2300. (B) Corresponding upward leaf roll (ULR) and swollen vein (SV) severity scores at four-day intervals from 12 to 36 dpi. (C) Log viral fold change at 20 and 36 dpi. Bars represent mean ± SD. Student’s t-test levels of significance: *, *p* < 0.05; **, *p* < 0.01.

Sequence differences present in the V2 coding region were selected as targets for generating full V2 sequence swap mutant chimeras (S1 Table) with the aim of investigating the influence of this region on disease symptom development in *N*. *benthamiana*. Plants infected with V30ΔV2-s (nt 139–489 swapped) displayed symptoms nearly identical to V30-infected plants, with no change in ULR symptom severity. Symptom recovery was observed as a decrease in mean SV severity between 24 and 36 dpi (Fig 4A and 4B). At 36 dpi, the mean SV severity score was significantly lower than that recorded for V30-infected plants. This apparent recovery in SV symptoms was associated with a substantial reduction in leaf surface rugosity when compared to plants infected with V30. Comparative analysis of viral load showed that plants infected with V30ΔV2-s had a corresponding significantly lower viral load than plants infected with V30 at 36 dpi (*p* = 0.032) (Fig 4C).

**Fig 5:**
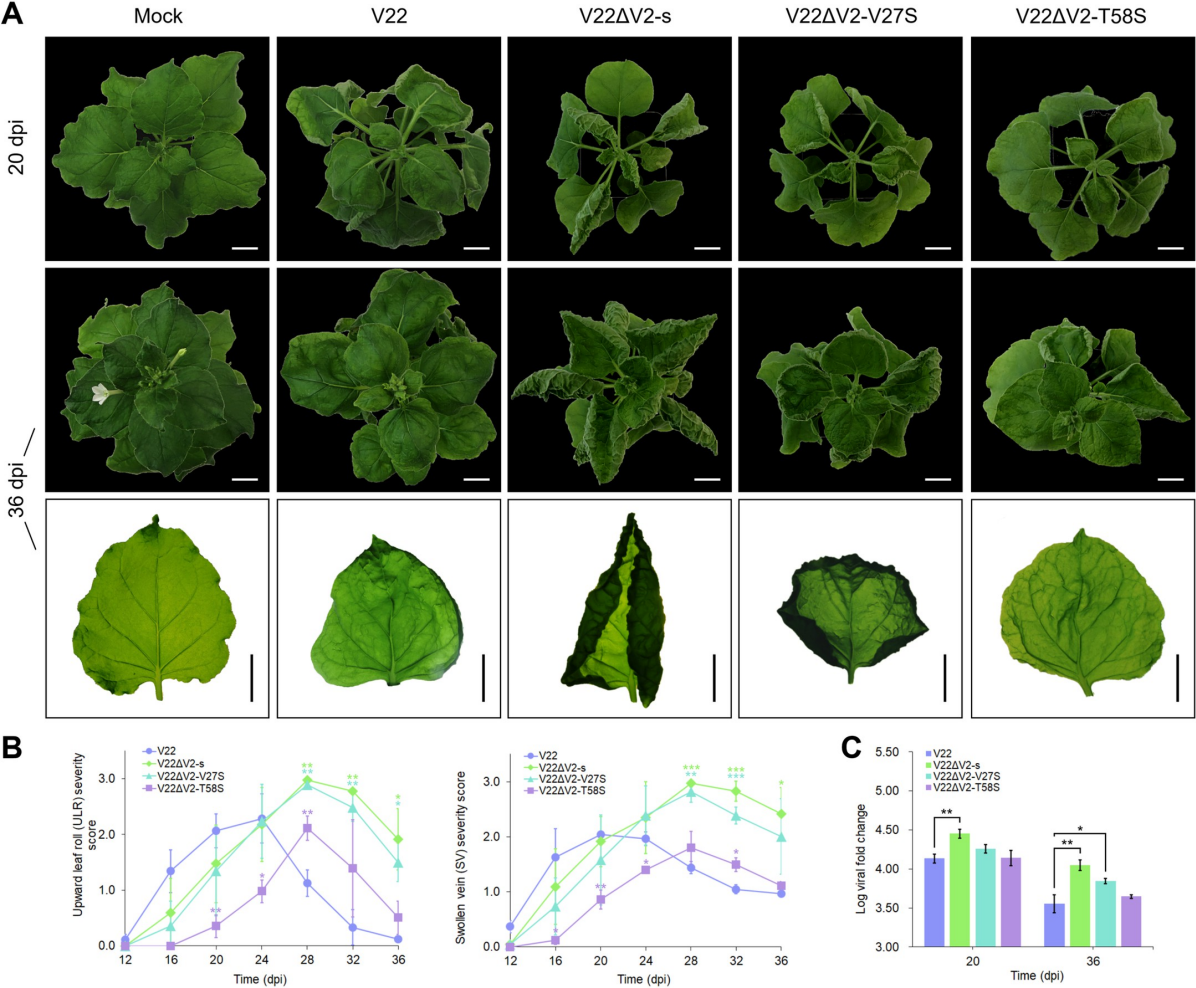
Single infection of *Nicotiana benthamiana* with V22 V2-swap mutant and V2 site-directed mutant infectious clones. (A) Symptoms in plants inoculated with V22, V22ΔV2-s, V22ΔV2-V27S, and V22ΔV2-T58S at 20 dpi (top row) and 36 dpi (middle and bottom rows). Bar = 2 cm. Mock inoculated with *Agrobacterium tumefaciens* C58C1 carrying empty pCAMBIA2300. (B) Corresponding upward leaf roll (ULR) and swollen vein (SV) severity scores at four-day intervals from 12 to 36 dpi. (C) Log viral fold change at 20 and 36 dpi. Bars represent mean ± SD. Student’s t-test levels of significance: *, *p* < 0.05; **, *p* < 0.01; ***, *p* < 0.001.

In contrast, plants infected with V22ΔV2-s (nt 138–488 swapped) displayed a very significant increase in both severity and duration of ULR and SV symptoms with a loss of V22-type recovery phenotype (Fig 5A and 5B). The broad upward rolling of the entire leaf edge resulted in severe leaf distortion. After 28 dpi mean symptom severity decreased but by 36 dpi both ULR and SV remained at peak severity levels seen in V22-infected plants. Relative viral load in plants infected with V22ΔV2-s was measured and a very significant increase in viral load was seen in mutant-infected plants compared to V22-infected plants at 20 dpi (*p* = 0.002) and 36 dpi (*p* = 0.005) (Fig 5C).

When generating V2 ORF swap mutants, swapping of the V2 coding region to obtain V30ΔV2-s and V22ΔV2-s resulted in additional aa changes to the proteins encoded by the V1 ORF and the two putative ORFs C5 and C6 which partially overlap with V2 (S7 Table). Site-directed mutagenesis was used to generate a V30 C6 deletion mutant (V30ΔC6-d) to truncate the V30 putative C6 ORF protein product by 35 aa resulting in a C6 protein identical in length to V22 C6 (108 aa). Plants agroinoculated with this mutant displayed mostly unchanged disease development with the exception of significantly higher SV symptom severity at late infection, although no significant changes in viral load were detected at either 20 or 36 dpi (S5 Fig).

Sequence differences in the well-characterised V2 protein were of particular interest, and site-directed mutagenesis was done to determine if aa sequence changes to the V22 V2 protein resulted in an altered disease phenotype or change in symptom recovery levels. Two residues were selected as targets to generate serine substitution mutants: Val27 present between H1 and H2, and Thr58 of the V2 PKC motif (Fig 1C). Serine substitution was applied since the aa sequence identity of these two residues at these positions was serine in V30 V2 (Fig 1C). Plants infected with V22ΔV2-V27S developed significantly increased ULR and SV symptoms compared to V22-infected plants with symptom severity peaking at 28 dpi and elevated ULR severity persisting by 36 dpi (Fig 5A and 5B). Symptom severity and progression was similar to that seen in *N*. *benthamiana* infected with V22ΔV2-s, and disease severity was not significantly different between V22ΔV2-s and V22ΔV2-V27S-infected plants at 36 dpi (Fig 5A and 5B). ULR severity in plants infected with V22ΔV2-V27S remained significantly higher than in V22-infected plants at 36 dpi whilst SV did not differ significantly by this timepoint (Fig 5A and 5B). Measurement of viral load relative to *GAPDH* showed that plants infected with V22ΔV2-V27S had a significantly higher viral load at 36 dpi than those infected with V22 (*p* = 0.039) (Fig 5C). A significant delay in ULR and SV symptom development was observed in plants infected with V22ΔV2-T58S (Fig 5A and 5B). Symptom severity peaked at 28 dpi but was not significantly different from peak severity levels seen in V22-infected plants. Recovery in both ULR and SV was seen by 36 dpi and disease severity was identical to that seen in V22-infected plants (Fig 5A and 5B). Viral load in V22ΔV2-T58S-infected plants did not differ significantly from V22-infected plants (Fig 5C).

### Evidence of bidirectional readthrough transcription in ToCSV-infected *N*. *benthamiana*

**Fig 6:**
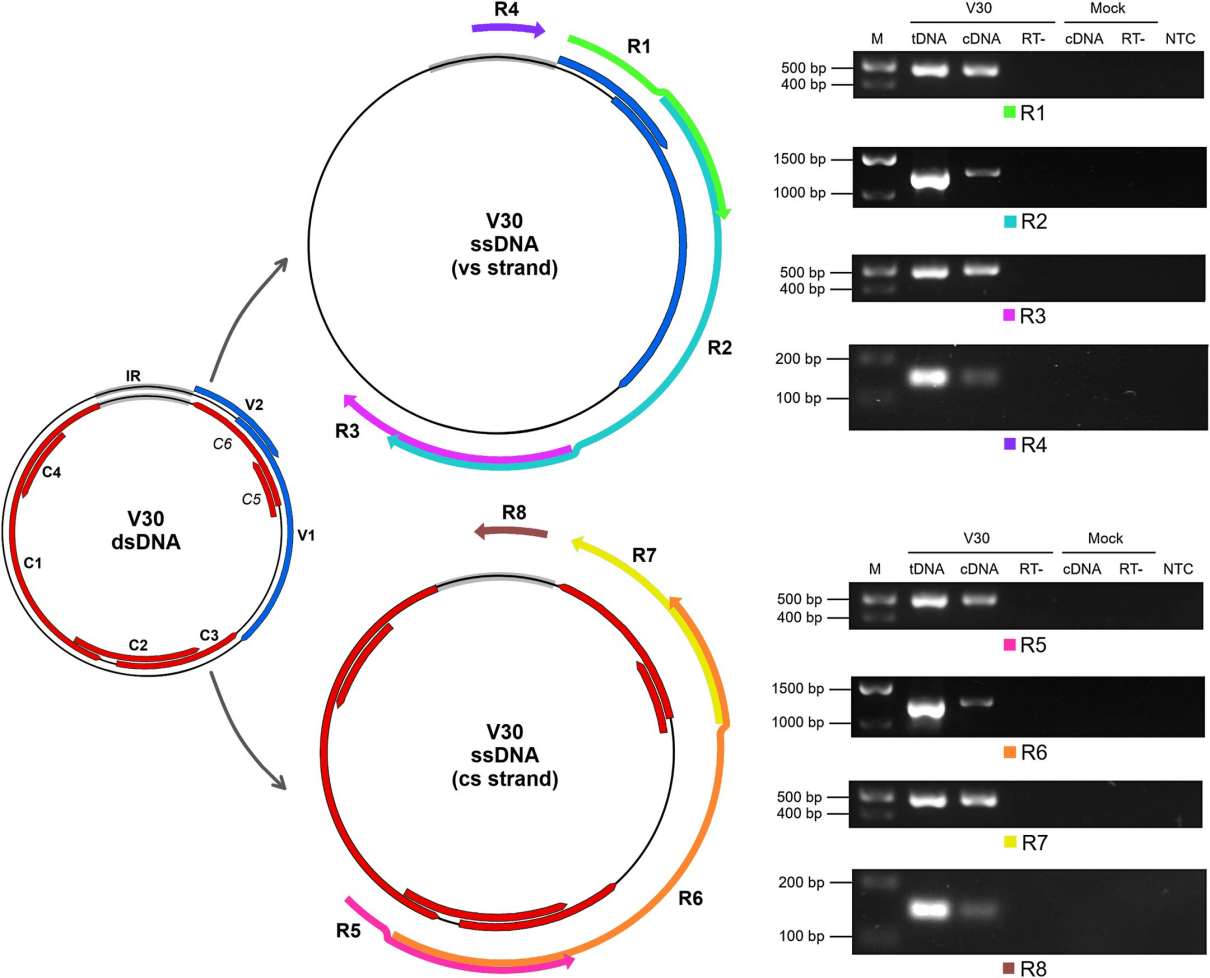
Identification of virus-derived RNA transcripts in V30-infected *Nicotiana benthamiana* using strand-specific RT-PCR. V30 dsDNA on the left with virion-sense (vs) ORFs in blue, complementary-sense (cs) ORFs in red, and IR in grey. Corresponding positions of virus-derived RNAs as RT-PCR products (R1-R8) depicted relative to V30 ssDNA vs and cs strands with 5′-3′ direction indicated. Agarose gel images of respective RT-PCR products shown on the right. M, DNA molecular weight marker; tDNA, PCR of total DNA extracted from plant inoculated with V30 (positive control); cDNA, RT-PCR of total RNA extracted from plants inoculated with V30 or mock; RT-, negative reverse transcriptase enzyme control; NTC, PCR no template control. Mock is RT-PCR of total RNA extracted from plant inoculated with *Agrobacterium tumefaciens* C58C1 carrying empty pCAMBIA2300.

To investigate the presence of long genome-derived RNA transcripts in virus-infected *N*. *benthamiana*, RT-PCR using strand-specific primers for cDNA synthesis was performed. All RT-PCR target products originating from both vs and cs strands were positively identified in RNA extracted from V30-infected plants and were mapped to the viral genome (Fig 6). The four vs strand-derived targets identified were: R1 spanning the V1/V2 ORF overlap, R2 spanning this overlap and extending substantially beyond the V1 stop codon, R3 mapping to a region of the vs not predicted to encode any proteins ≥60 aa in length when following standard code, and R4 which was mapped to the IR crossing the Ori. The four cs strand-derived targets included: R5 spanning the C1/C2 ORF and C2/C3 ORF overlaps, R6 spanning the C1 ORF 3’ region to putative C6 ORF, R7 mapping to the region encoding the full putative C5 and C6 ORFs, and R8 spanning the IR crossing the Ori (Fig 6). The RT-PCR target product sizes and the relative V30 genome nt positions are shown in Table 1. Two additional RT-PCR target products (R9 and R10) were identified in V22-infected plants confirming the presence of RNA transcripts derived from the IR crossing the Ori on both vs and cs strands (S6 Fig).

**Table 1:** RT-PCR product targets identified in this study.

RT-PCR product	Product size (bp)	Relative V30 nt position (5’-3’)
R1	494	142–635
R2	1243	367–1609
R3	504	1231–1734
R4	137	2722–92
R5	504	1734–1231
R6	1243	1609–367
R7	494	635–142
R8	137	92–2722

## Discussion

### The coding capacity of ToCSV is expanded to include two novel putative ORFs

In the present study we investigated the genomic coding capacity and in vivo infectivity of two ToCSV sequence variants sharing 96% nt sequence identity. Using ORF finder, we identified two putative ORFs, which we designated C5 and C6 based on sequence comparison with other putative begomovirus ORFs identified by BLAST analysis. Short putative ORFs present in the genome of monopartite geminiviruses (and DNA-A of bipartite begomoviruses) have recently gained increasing attention for their role in facilitating viral infection [20, 61–66]. Through in vivo ribosome profiling of tomato yellow leaf curl Thailand virus, a series of novel translation initiation sites (TISs) were identified including two which result in the translation of distinct functional isoforms of AV2. The translation of a novel “hidden” ORF nested within BV1 on DNA-B (termed BV2) encoding a functional protein was also demonstrated [67]. Although no satellite molecules have been positively identified in association with ToCSV [11, 16], the βC1 and novel βV1 proteins encoded by begomovirus betasatellites have recently been shown to exert several important functions related to pathogenicity, virus-host interactions, and suppression of host antiviral defense [68–71]. Taken together, these ground-breaking studies highlight the need to further characterize the coding capacity of geminivirus genomes as well as their associated satellites to advance the understanding of the complex mechanisms involved in their increased proliferation. Whilst we did not confirm the expression of the putative ToCSV ORFs in vivo, a cs strand-derived transcript spanning the putative C5 and C6 ORFs was identified using RT-PCR with cs strand-specific primers. To validate the existence of the C5 and C6 ORFs, additional experiments are necessary in future. These include using 5’ and 3’ rapid amplification of cDNA ends (RACE) assays to define their transcripts, and western blots with anti-C5/C6 antibodies to confirm in vivo translation. Mapping of putative C5/C6 promoter regions will also provide more insight into the expression of these ORFs in ToCSV-infected plants.

The C5/AC5 protein has been shown to function as a pathogenicity determinant and a potent inhibitor of antiviral silencing pathways in begomovirus-infected *N*. *benthamiana* [19, 20, 66]. The first major study of several OW and NW geminivirus C5/AC5 proteins by Li et al. [19], described two recognised C5/AC5 protein domains: ‘Gemini AC5-1’ (pfam04807) and ‘Gemini AC5-2’ (pfam08464). Begomovirus C5/AC5 proteins containing the Gemini AC5-1 domain have been demonstrated to localise to the nucleus [62], act as pathogenicity determinants, and suppress or reverse TGS and PTGS [19, 20, 66] in *N*. *benthamiana*. The C5 protein of ToCSV lacks the Gemini AC5-1 domain, whilst the function of the Gemini AC5-2 domain (present in ToCSV C5) is unknown and requires further study.

Based on our investigation of several OW and NW begomovirus genomes it is apparent that the C6/AC6 ORF is less common than C5/AC5 (data unpublished). In the case of ToCSV, the C6 ORF is exceptionally large, encoding a putative protein with a higher molecular weight than V2 in the case of V30 C6. We compared ToCSV C6 protein sequences with that of a series of proteins encoded by small non-canonical ORFs recently described from the genome of TYLCV by Gong et al. [62]. It was determined that the 62 C-terminal residues of V22 C6 shared 79% sequence identity with a protein encoded by TYLCV “ORF3” that was shown by the authors to localise to the mitochondria in *N*. *benthamiana* when expressed as a GFP-fusion. Both of our ToCSV variant C6 proteins were predicted to contain signal peptides and a transmembrane domain in the N-terminal region using the Phobius webserver [72] (S7 Fig). Interestingly, Gong et al. [62] did not identify a transmembrane domain in their ORF3-encoded protein and theirs also lacked the N-terminal region containing the transmembrane domain present in the V30 and V22 C6 proteins. Expression of the tomato leaf curl China virus (ToLCCNV) C6 was recently confirmed and the C6 protein was shown to contain residues necessary for directing protein localisation to the mitochondria [65]. The authors found that C6 residues 30–97 were responsible for mediating this localisation. BLAST analysis in this current study showed that this region aligned with V30 C6 residues 77–143, suggesting that mitochondrial localisation of C6 may be altered in V22 C6 since this protein lacks the 35 C-terminal residues of the C6 region implicated in mitochondrial localisation. We determined through deletion of the 35 C-terminal residues of V30 C6 that this region is not required for viral replication in *N*. *benthamiana* but may be implicated in modulating SV symptom severity via an unknown mechanism. Interestingly, Wang et al. [65] demonstrated through PVX-vector expression of ToLCCNV C6 and mutagenesis to generate a C6-null mutant that C6 is not a pathogenicity determinant in *N*. *benthamiana* and is not required for disease symptom expression.

It is possible that the C6 protein may have a repertoire of accessory functions which could serve to fine-tune crucial infection events occurring at different regions within the cell. Such events driven primarily by the more conserved canonical proteins may be assisted in part by the activity of non-canonical proteins such as C6. Further testing is necessary to determine if the observed variability in C6 protein length enables diversity in protein function. The C6 proteins of ToCSV contained two to three internal methionine residues further supporting our hypothesis that multiple forms of this protein may exist. Ribosome profiling in future could prove useful in identifying the associated TISs. In our study, additional mutagenesis experiments designed to mutate several additional C6 regions of interest (including extension of the V22 C6 C-terminus) were severely restricted by the presence of overlapping V1, V2 and C5 ORFs. PVX-vector expression of wild type and mutant C6 proteins should be conducted to determine if ToCSV C6 is a pathogenicity determinant and to identify key sequence motifs that could serve critical function in mitochondrial localisation.

### The ToCSV 3’ IR is a symptom phenotype determinant

The present study is the first to report on the infectivity of two infectious clone constructs of ToCSV in *N*. *benthamiana*, a well-accepted experimental host used in plant virus studies [73]. In tomato ‘Rooikhaki’, a begomovirus-susceptible South African cultivar, symptoms induced by V30 and V22 are identical except V22-infected plants display a significantly milder symptom phenotype [16]. In *N*. *benthamiana* however, the severity of ULR and SV symptom phenotypes induced by V30 and V22 differed significantly and symptom recovery was observed plants infected with V22 but not V30. In the present study we sought to determine the precise sequence differences responsible for the distinct symptom phenotypes induced in *N*. *benthamiana*. Sequence differences of only a few nt in other geminivirus isolates have been shown to result in significantly altered disease symptom phenotypes and viral accumulation [74–77]. Using 3’ IR sequence swap mutant chimeras, we deduced that the TACE-containing sequence to the right of the TATA box played a crucial role in symptom phenotype determination but not in V22-specific recovery in infected *N*. *benthamiana*. The short variable 3’ IR region upstream of the TATA box promoter (nt 61–108 in V30/V22) did not contain any known *cis*-acting elements. We inoculated *N*. *benthamiana* with additional IR-swap mutants (nt 61–108 swapped: V30ΔIR-sl and V22ΔIR-sl) and did not observe significant changes to disease symptom phenotypes when compared with plants inoculated with V30 or V22 (S8 Fig). Using the chimeric genome approach, An et al. [78] determined that swapping the IR of DNA-B of pepper yellow leaf curl Thailand virus (PYLCThV) with that of tomato yellow leaf curl Kanchanaburi virus (TYLCKaV) conferred to the mutant PYLCThV the ability to infect tomato. Although the authors showed that the presence of the IR of TYLCKaV DNA-B was a prerequisite for the ability of their mutant chimeras to infect tomato, they did not identify precise IR sequence regions that are responsible for determining infectivity.

The TACE was previously described as a putative *cis*-acting element in the 3’ IR of both OW and NW begomoviruses [60]. We determined that the TACE of V30 did not conform to the TACE consensus sequence or arrangement typical of other OW begomoviruses as described by Cantú-Iris et al. [60]. To our knowledge, this is the first report of these atypical features in the TACE of any OW begomovirus. Although Cantú-Iris et al. [60] did not experimentally determine the function of the TACE, the authors suggested that the TACE is involved in “TrAP-mediated derepression” of the V1 ORF in infected phloem tissue. It may be worthwhile to generate additional V30 IR mutants which contain either the TACE N_7_ central spacer region of V22 or have a deletion of the additional spacer sequence (GGGAA) between the TATA box and the TACE left arm. Such targeted mutation could provide clues as to which of these specific TACE features are responsible for the differing symptom phenotypes and what role these features may exert on transcription regulation activity. The unusual TACE of V30 makes it a prime candidate for additional experiments to elucidate the role of this novel element in begomovirus-induced disease.

Since the 3’ IR contains the promoter regions driving expression of downstream vs genes, as well as *cis*-acting elements involved in regulating their transcription [23, 24] any sequence differences in this region may result in differential expression of vs ORFs in V30- and V22-infected plants. Quantitative RT-PCR should be conducted to determine if V1/V2 transcript levels differ between the two wild types as well as selected IR-swap mutants. In addition, infectivity trials in susceptible tomato are necessary to determine if modification of the IR region alters the symptom phenotype in the crop host.

### The ToCSV V2 coding region is involved in modulating disease severity

The role of sequence differences present in the V2 coding region was also investigated through agroinoculation of *N*. *benthamiana* with two full-length canonical V2 ORF swap mutants. Plants infected with these mutants did not display altered symptom phenotypes. Instead, the key difference observed was a significant change in disease severity and altered recovery. The begomovirus C4 protein has recently been shown to exert significant influence on the development and severity of leaf curling symptoms in both *N*. *benthamiana* [79] and tomato [80]. It has been determined that the V2 protein of TYLCV can enhance the accumulation of the C4 protein in *N*. *benthamiana* independent of its ability to suppress RNA silencing [81] and therefore sequence differences in the V2 protein of ToCSV may serve to modulate this newly-discovered function of V2. Further experiments are necessary to determine whether the potential C4-enhancing activities of V30 and V22 V2 proteins differ significantly. The results obtained with our V2 ORF swap chimeras confirmed our assertion that a region outside of the IR was responsible for modulating the recovery phenotype observed in V22-infected plants. Although these observed changes could be due to changes in the aa sequence of proteins encoded by the overlapping ORFs present in the V2 coding region, changes in nt sequence identity should also be taken into consideration. The region of V1/V2 overlap is a known “hotspot” that acts as a major source of vsRNA in both TYLCV and TYLCSV [21, 82]. Recovery in geminivirus-infected plants has been linked to the presence of virus-derived siRNAs involved in RNA silencing pathways [83, 84]. Differences in pathogenicity or infectivity observed in this study between variant isolates may thus be mediated by the differing vsRNA profiles. Differing putative promoter, *cis*-acting element, and iteron sequences present in this region could also be exerting an effect. Although promoter elements have not yet been mapped for ToCSV, sequences with promoter activity have been found within coding regions of other begomoviruses [62, 85, 86]. Promoter mapping of the two variant sequences may uncover additional sequence features which are implicated in differentiating pathogenicity through their varied activities.

The critical role that the V2/AV2 protein of both mono- and bipartite geminiviruses plays in viral infection and pathogenicity has been reviewed extensively [24, 87, 88]. To examine whether specific aa sequence differences present in the N-terminal region of the V2 protein are involved in regulating recovery, targeted mutagenesis of the V22 V2 was done. Serine substitution of V2 Val27 resulted in a significant increase in symptom severity and reduction in disease recovery comparable to that induced by V22ΔV2-s. Viral load was significantly increased at late infection timepoint but not at peak infection (as was the case in V22ΔV2-s-infected plants). Taken together these results imply that additional sequence differences contribute to the significantly increased viral load seen at peak infection in plants infected with the full V2 ORF swap mutant. Although no published information exists regarding the specific function of V2 aa 27, this residue is flanked on either side by hydrophobic domains H1 and H2 containing residues which have been shown to be necessary for pathogenicity and efficient RNA silencing activity in other geminiviruses [34, 89]. Mutation of V30 V2 is necessary to determine if substitution of V2 Ser27 with valine (to V22 V2 sequence identity) will consequently reduce pathogenicity and introduce a recovery phenotype as was observed in plants infected with V30ΔV2-s. The specific function that V2 aa 27 exerts in RNA silencing suppression pathways should be investigated further.

We observed that serine substitution of V2 Thr58 resulted in delayed symptom development. V2 aa positions 58 to 60 constitute the PKC motif which has been reported to have a consensus sequence of Thr-X-Arg, where X is any residue [31, 33, 35]. Alanine substitution of the conserved PKC motif residues previously showed that these residues are required for the subcellular localisation, pathogenicity-enhancing, and RNA silencing suppression activities of several geminivirus V2 proteins [34, 35, 89]. Although threonine has been defined as a conserved residue in the PKC motif, a serine at this position has been reported in selected cotton-infecting begomovirus isolates [31, 33, 34]. The effect of substituting the consensus threonine of the PKC motif with a non-consensus serine residue has not been previously investigated in other geminivirus V2 proteins. Our observations suggest that although serine substitution of the threonine does not impair viral replication, the delay in symptom development may be due to possible interference with V2 function but further experiments are necessary to confirm this. It is not known what, if any, positive influence a serine at V2 aa position 58 exerts on pathogenicity in V30 infection. No delay in symptom development was seen in V30-infected plants (when compared with plants infected with V22), which implies that the non-consensus serine present in V30 PKC motif may not be detrimental to V2 function in V30 infection. This atypical serine-containing PKC motif may result in modified V2 function that is advantageous to V30 but not V22, although additional experiments are necessary to confirm this. The results from our mutagenesis experiments indicate that the aa sequence identity of the V2 protein plays a crucial role in the differing ToCSV variant pathologies. By identifying the precise functions of selected aa present in the V2 proteins of V30 and V22, the mechanism of recovery could be better understood.

### Implications of bidirectional readthrough transcription of ToCSV in infected *N*. *benthamiana*

In this study we detected the presence of long viral RNA transcripts derived from both vs and cs strands of coding and non-coding regions in ToCSV-infected *N*. *benthamiana*. The dsDNA replicative form of the geminivirus is known to act as a template for bidirectional transcription of vs and cs ORFs driven by promoter sequences within the IR [90–93]. Through transcript mapping of several begomoviruses, three major polycistronic transcripts have been previously identified: one derived from the vs strand (~1.0 kb spanning V1, V2) and two derived from the cs strand (~1.7 kb spanning C1, C2, C3, C4; and ~0.7 kb spanning C2, C3) [90, 91, 94, 95]. The 3’ termini of the polyadenylated vs and cs transcripts slightly overlap where the V1 and C3 ORFs terminate [90, 94, 96]. In the present study, we mapped two long transcripts (R2 and R6) extending substantially past the recognised 3’ termini of the vs and cs strand-derived polycistrons. Additionally, we identified a vs-strand derived transcript overlapping the region of the C1/C2/C3 ORF overlap as well as a cs strand-derived transcript spanning the putative C5/C6 ORFs. Taken together, these findings constitute evidence of bidirectional readthrough transcription in ToCSV-infected *N*. *benthamiana*. This phenomenon has been linked to siRNA biogenesis implicated in downstream RNA silencing pathways in plants infected with other geminiviruses [82, 84, 93, 97–99].

Using RT-PCR, we also demonstrated the presence of transcripts ~140 nt in length derived from both strands of the IR crossing the Ori in V30- and V22-infected *N*. *benthamiana*. The presence of these overlapping transcripts strongly supports prior findings which indicate that the IR of geminiviruses acts as a potential source of dsRNA which may be processed further to generate viral siRNAs for RNA silencing pathways [21, 93, 100, 101], although the coding regions of geminiviruses tend to yield more significant amounts of siRNAs [93]. Viral siRNAs derived from the IR of TYLCV have been shown to act as virulence effectors by directing the silencing of long noncoding RNAs originating from the tomato host genome [101]. Although we did not characterise the identified IR transcripts further, deep sequencing of V30 and V22-infected plants would allow for comparative analysis of IR-derived viral siRNA profiles.

## Conclusions

Using ORF finder we identified two putative ORFs, C5 and C6, and provided evidence for possible transcription of these two ORFs. Additional experiments to validate their expression and determine their function in ToCSV-infected plants are warranted. Upon consideration of all the results obtained through inoculation of the model host *N*. *benthamiana* with two sequence variants of ToCSV, we conclude that nt sequence differences present in the 3’ IR sequence between the TATA box and the V2 start codon containing the TACE are responsible for the symptom phenotype determination; whilst sequence differences present in the V2 coding region are responsible for the V22-specific recovery phenotype. Using site-directed mutagenesis, we provide evidence that V2 aa 27 specifically exerts a significant influence on the differing symptom severity and symptom recovery levels and appears to act as a key residue in V2 function. We provide confirmation of bidirectional readthrough transcription in ToCSV-infected *N*. *benthamiana* with several potential regions of interest having been identified as sources of RNA transcripts. The mechanisms of their downstream processing and their activity in vivo should be investigated further. Whilst additional experiments are necessary particularly in the tomato host, our study serves to improve understanding of the complex dynamics involved in ToCSV pathogenicity with the aim of developing suitable strategies aimed at the surveillance and control of this pathogen in South Africa.

## Supporting information

S1 Fig *Nicotiana benthamiana* leaf edge zone demarcations used in upward leaf roll (ULR) symptom severity scoring system. Seven leaf zones are numbered.
(TIF)

S2 Fig qPCR primer testing. A, standard curves generated for internal control *glyceraldehyde 3-phosphate dehydrogenase* (*GAPDH*) and viral V2 ORF (V2) primers using a 10-fold dilution series of 100 ng total DNA extracted from *Nicotiana benthamiana* inoculated with ToCSV V30. B, corresponding qPCR melting curves showing single peaks for both products.
(TIF)

S3 Fig Pairwise V30 and V22 putative protein aa sequence alignment. A, C5 and B, C6 protein aa sequence alignment generated using PRALINE with colour key indicating aa conservation. Conserved C5 ‘Gemini AC5-2’ domain (pfam08464) boxed in black. Hyphens indicate alignment-generated gaps. Asterisks indicate sequence conservation.
(TIF)

S4 Fig Partial intergenic region (IR) viral motif/regulatory element sequence alignment. TATA box and TATA-associated composite element (TACE) to V2 start codon (italicised) indicated for ToCSV-V30, ToCSV-V22, and selected African tomato-infecting monopartite begomoviruses sharing ≥78% nt sequence identity with ToCSV. Highlighted colours indicate motif consensus sequence present: TATA highlighted green, TACE left and right arms highlighted yellow, TACE spacer region highlighted orange. Hyphens indicate alignment-generated gaps, ellipsis indicates spacer sequence with nt number shown. For details of all isolate sequences, refer to S3 Table.
(TIF)

S5 Fig Single infection of *Nicotiana benthamiana* with V30 C6 deletion mutant infectious clone. (A) Upward leaf roll (ULR) and swollen vein (SV) severity scores obtained for plants inoculated with V30, and V30ΔC6-d at four-day intervals from 12 to 36 dpi. (B) Log viral fold change at 20 and 36 dpi. Bars represent mean ± SD. Student’s t-test levels of significance: *, *p* < 0.05; **, *p* < 0.01.
(TIF)

S6 Fig Identification of virus-derived RNA transcripts in V22-infected *Nicotiana benthamiana* using strand-specific RT-PCR. Partial linearised V22 dsDNA on the left with virion-sense (vs) V2 ORF start in blue, complementary-sense (cs) C1 ORF start in red, and IR in grey. Corresponding positions of virus-derived RNAs as RT-PCR products (R9 and R10) depicted relative to V22 dsDNA vs and cs strands with 5′-3′ direction indicated. Relative V22 nt positions indicated at 5’ and 3’ ends. Agarose gel images of respective RT-PCR products shown on the right. M, DNA molecular weight marker; tDNA, PCR of total DNA extracted from plant inoculated with V22 (positive control); cDNA, RT-PCR of total RNA extracted from plants inoculated with V22 or mock; RT-, negative reverse transcriptase enzyme control; NTC, PCR no template control. Mock is RT-PCR of total RNA extracted from plant inoculated with *Agrobacterium tumefaciens* C58C1 carrying empty pCAMBIA2300.
(TIF)

S7 Fig Prediction of transmembrane domains and signal peptides from the aa sequence of ToCSV putative C6 proteins. Predicted location of transmembrane domains and signal peptides for (A) V30 C6, and (B) V22 C6. Prediction outputs generated using the Phobius webserver.
(TIF)

S8 Fig Single infection of *Nicotiana benthamiana* with V30 and V22 intergenic region (IR)-swap mutant infectious clones. Upward leaf roll (ULR) and swollen vein (SV) severity scores obtained for plants inoculated with (A) V30, and V30ΔIR-sl and (B) V22, and V22ΔIR-sl at four-day intervals from 12 to 36 dpi. Bars represent mean ± SD. Student’s t-test levels of significance: *, *p* < 0.05.
(TIF)

S9 Fig Original gel images used to generate Fig 6. (PDF)

S1 Table Description of sequence changes made to generate ToCSV variant IR and V2 sequence swap mutants. (DOCX)

S2 Table Primers used in this study. (DOCX)

S3 Table Monopartite begomovirus isolates used in this study. (DOCX)

S4 Table Upward leaf roll (ULR) symptom severity scoring system for *Nicotiana benthamiana*. (DOCX)

S5 Table Swollen leaf vein (SV) symptom severity scoring system for *Nicotiana benthamiana*. (DOCX)

S6 Table Predicted ORFs for ToCSV V30 and V22 genomes encoding proteins ≥60 aa in length. (DOCX)

S7 Table Predicted viral protein changes for V30ΔV2-s and V22ΔV2-s mutants following standard code. (DOCX)

S1 Dataset Upward leaf roll (ULR) and swollen vein (SV) symptom severity scores obtained for virus-infected *Nicotiana benthamiana*. (XLSX)

S2 Dataset Viral load in infected *Nicotiana benthamiana* determined relative to *glyceraldehyde 3-phosphate dehydrogenase* (*GAPDH*) using qPCR. (XLSX)

S1 Raw images (PDF)
