# Supplementary figures and images for: Differences in the 3’ intergenic region and the V2 protein of two sequence variants of tomato curly stunt virus play an important role in disease pathology in Nicotiana benthamiana
Source: PLoS One. 2023 May 23;18(5):e0286149. doi: 10.1371/journal.pone.0286149 (PMC10205009; doi:10.1371/journal.pone.0286149)

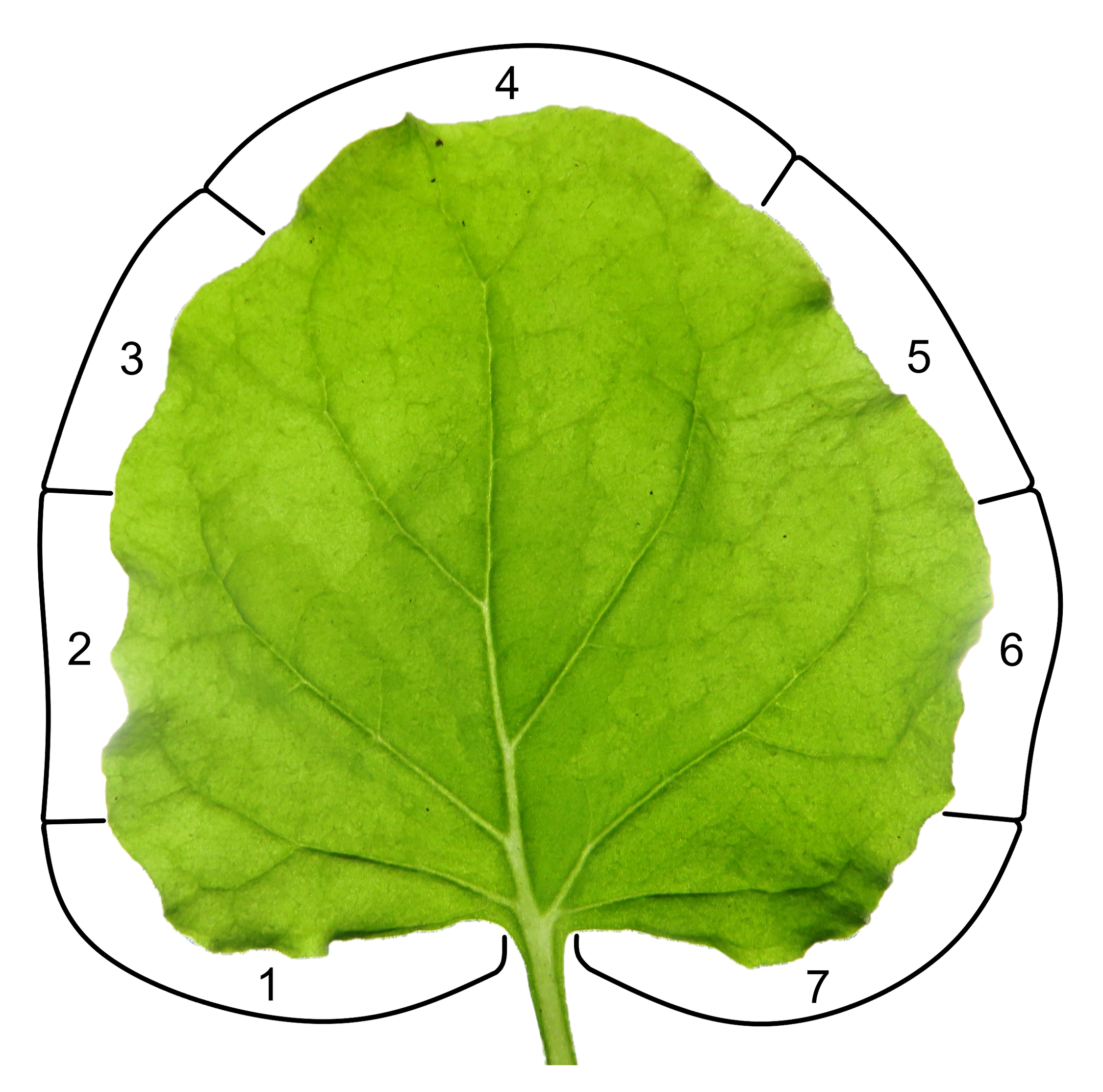

Supplement: S1 Fig — Seven leaf zones are numbered. (TIF) [file pone.0286149.s001.tif]

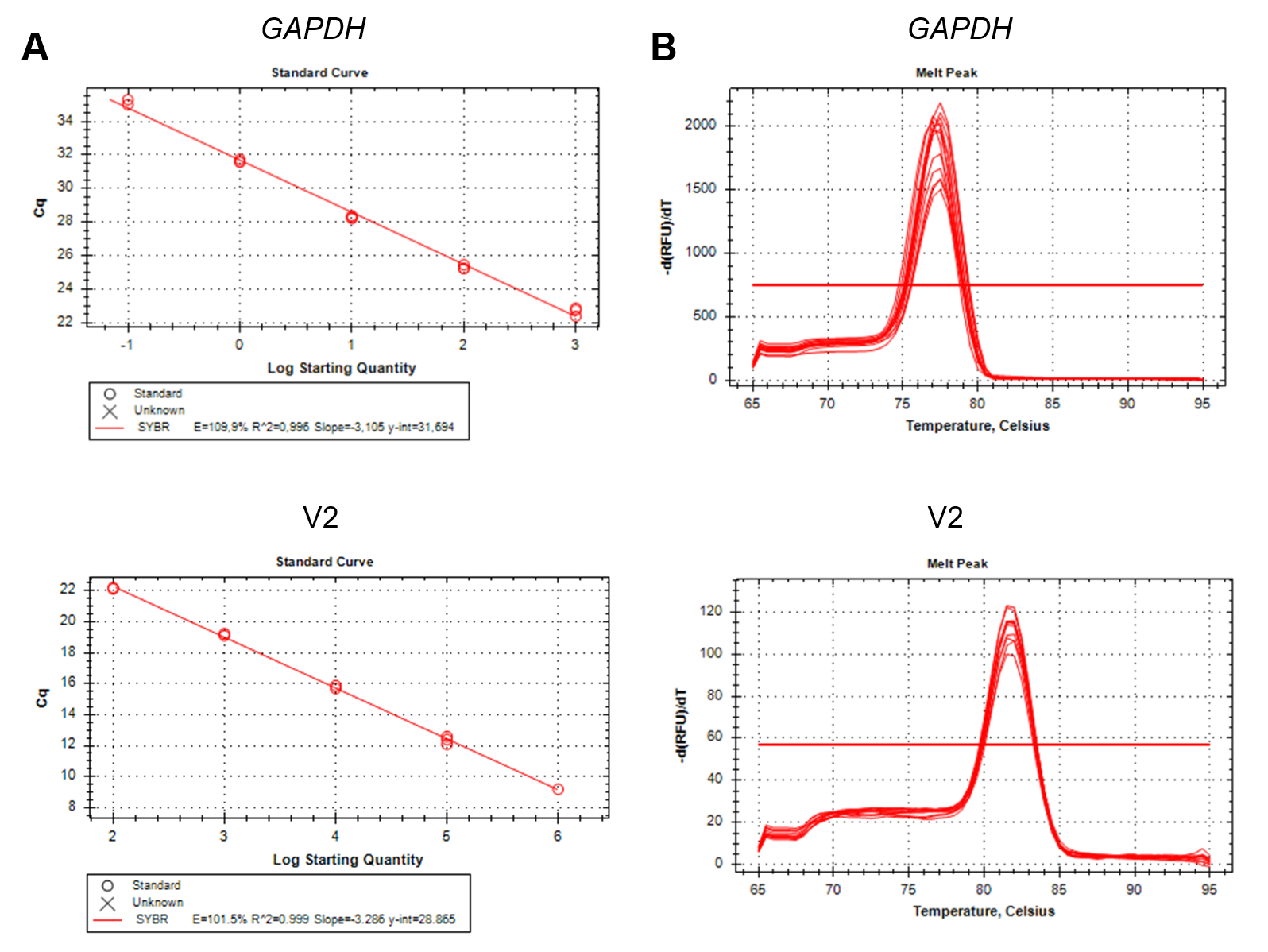

Supplement: S2 Fig — A, standard curves generated for internal control glyceraldehyde 3-phosphate dehydrogenase (GAPDH) and viral V2 ORF (V2) primers using a 10-fold dilution series of 100 ng total DNA extracted from Nicotiana benthamiana inoculated with ToCSV V30. B, corresponding qPCR melting curves showing single peaks for both products. (TIF) [file pone.0286149.s002.tif]

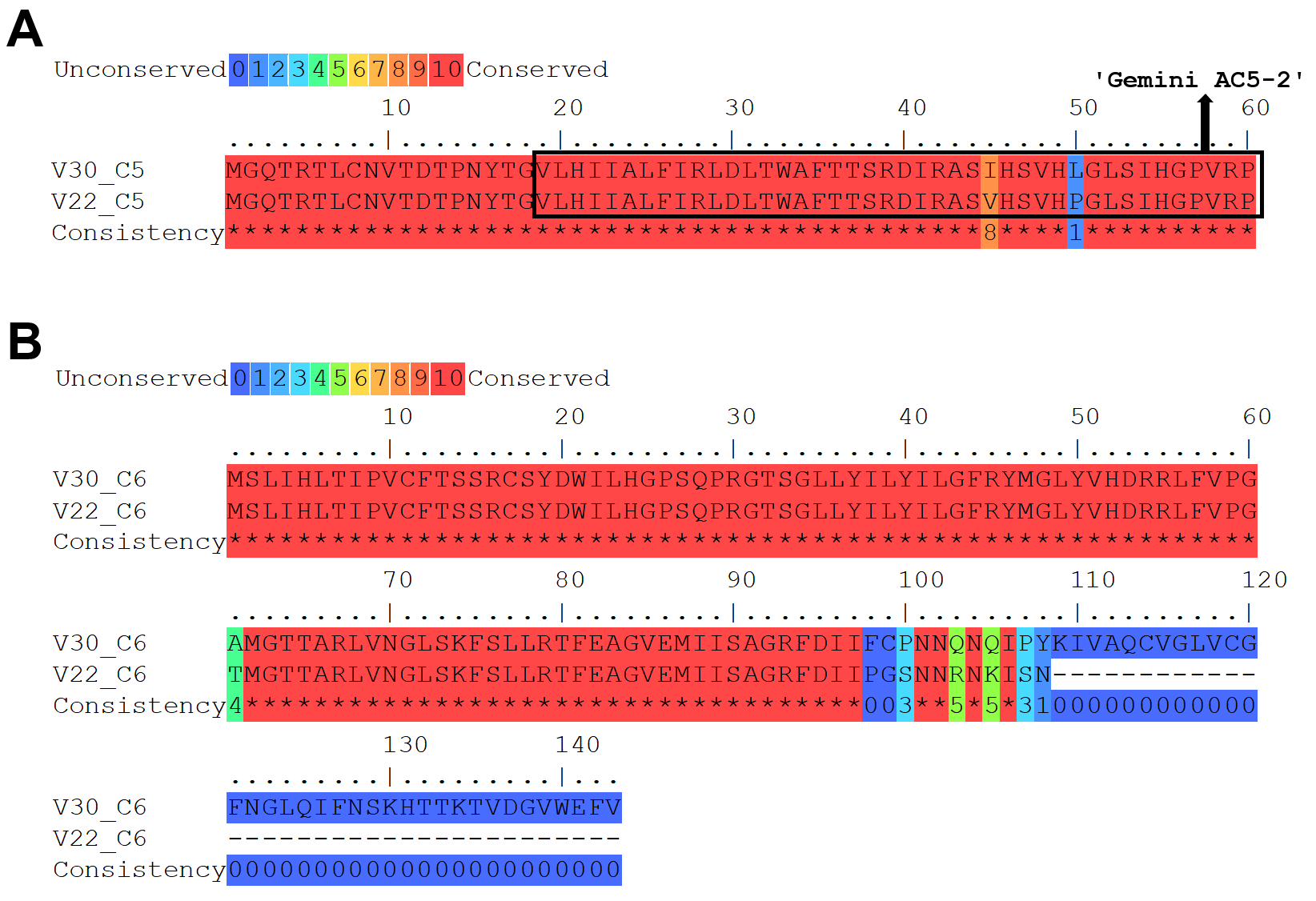

Supplement: S3 Fig — A, C5 and B, C6 protein aa sequence alignment generated using PRALINE with colour key indicating aa conservation. Conserved C5 ‘Gemini AC5-2’ domain (pfam08464) boxed in black. Hyphens indicate alignment-generated gaps. Asterisks indicate sequence conservation. (TIF) [file pone.0286149.s003.tif]

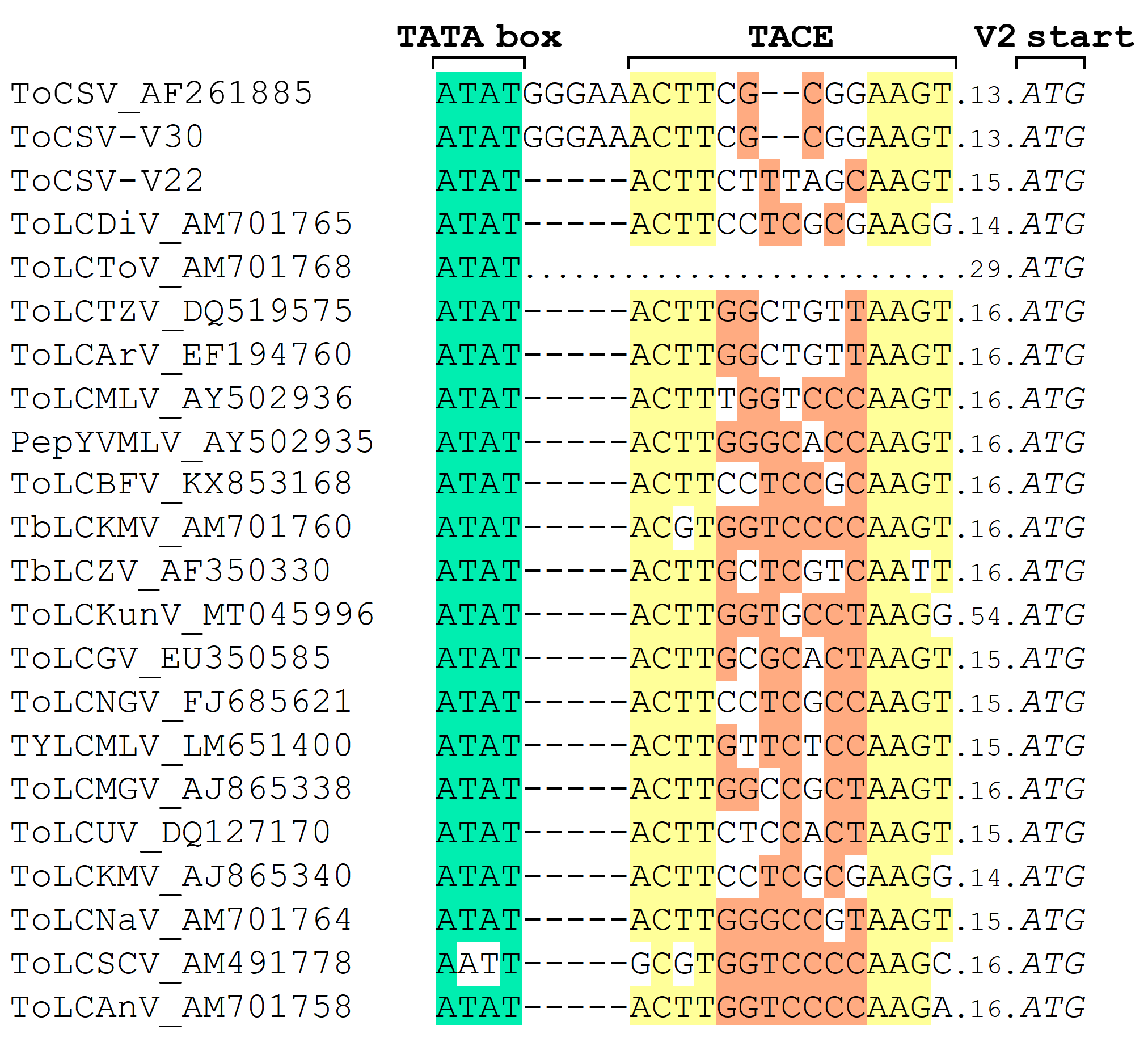

Supplement: S4 Fig — TATA box and TATA-associated composite element (TACE) to V2 start codon (italicised) indicated for ToCSV-V30, ToCSV-V22, and selected African tomato-infecting monopartite begomoviruses sharing ≥78% nt sequence identity with ToCSV. Highlighted colours indicate motif consensus sequence present: TATA highlighted green, TACE left and right arms highlighted yellow, TACE spacer region highlighted orange. Hyphens indicate alignment-generated gaps, ellipsis indicates spacer sequence with nt number shown. For details of all isolate sequences, refer to S3 Table. (TIF) [file pone.0286149.s004.tif]

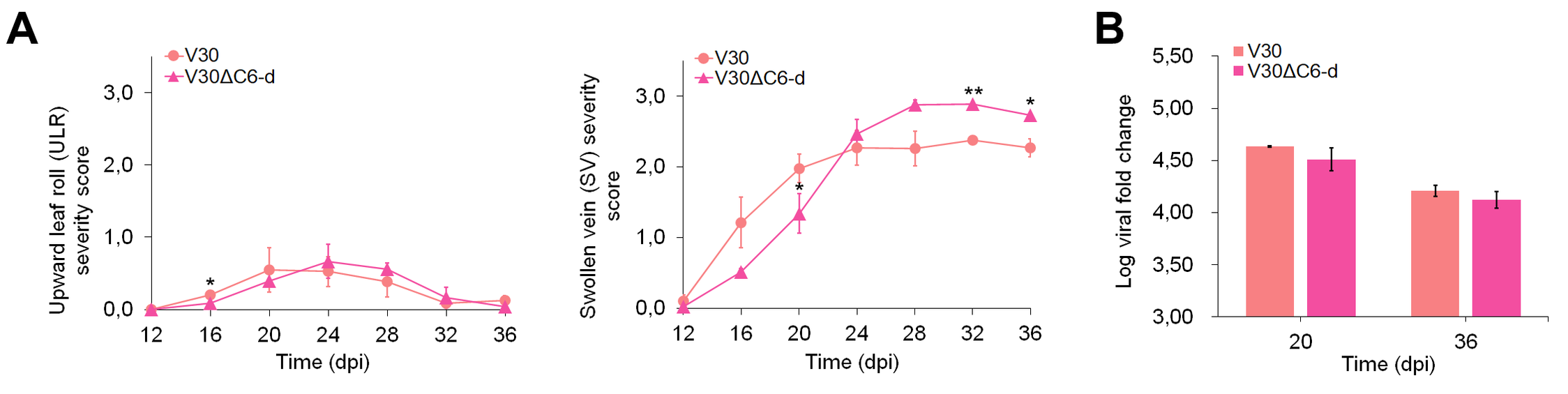

Supplement: S5 Fig — (A) Upward leaf roll (ULR) and swollen vein (SV) severity scores obtained for plants inoculated with V30, and V30ΔC6-d at four-day intervals from 12 to 36 dpi. (B) Log viral fold change at 20 and 36 dpi. Bars represent mean ± SD. Student’s t-test levels of significance: *, p < 0.05; **, p < 0.01. (TIF) [file pone.0286149.s005.tif]

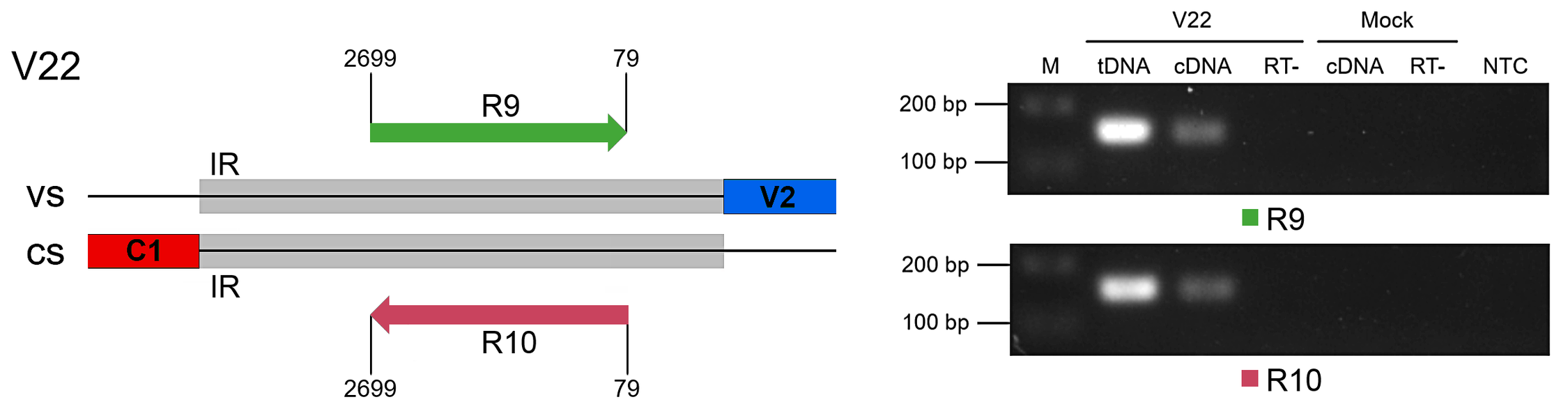

Supplement: S6 Fig — Partial linearised V22 dsDNA on the left with virion-sense (vs) V2 ORF start in blue, complementary-sense (cs) C1 ORF start in red, and IR in grey. Corresponding positions of virus-derived RNAs as RT-PCR products (R9 and R10) depicted relative to V22 dsDNA vs and cs strands with 5′-3′ direction indicated. Relative V22 nt positions indicated at 5’ and 3’ ends. Agarose gel images of respective RT-PCR products shown on the right. M, DNA molecular weight marker; tDNA, PCR of total DNA extracted from plant inoculated with V22 (positive control); cDNA, RT-PCR of total RNA extracted from plants inoculated with V22 or mock; RT-, negative reverse transcriptase enzyme control; NTC, PCR no template control. Mock is RT-PCR of total RNA extracted from plant inoculated with Agrobacterium tumefaciens C58C1 carrying empty pCAMBIA2300. (TIF) [file pone.0286149.s006.tif]

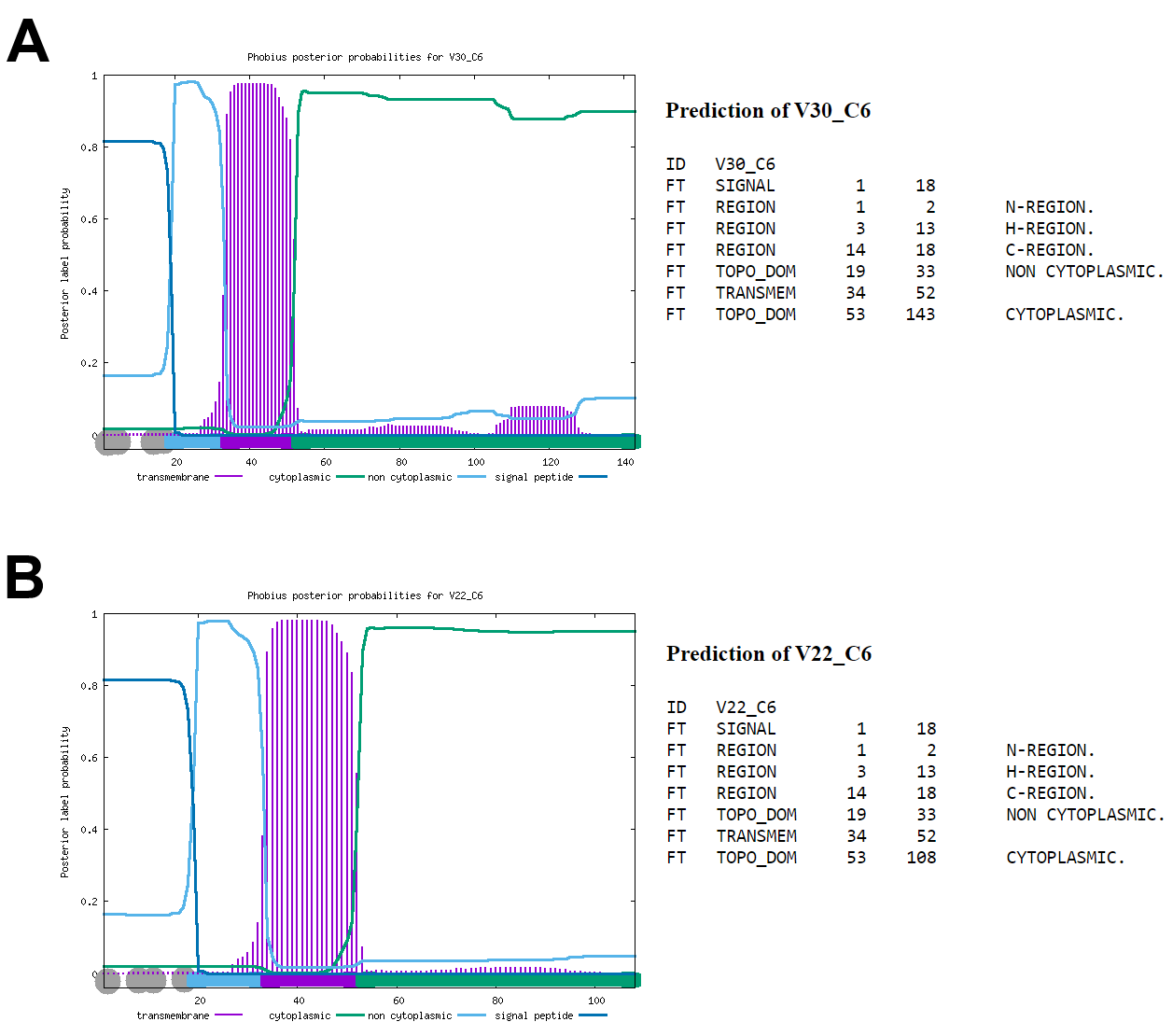

Supplement: S7 Fig — Predicted location of transmembrane domains and signal peptides for (A) V30 C6, and (B) V22 C6. Prediction outputs generated using the Phobius webserver. (TIF) [file pone.0286149.s007.tif]

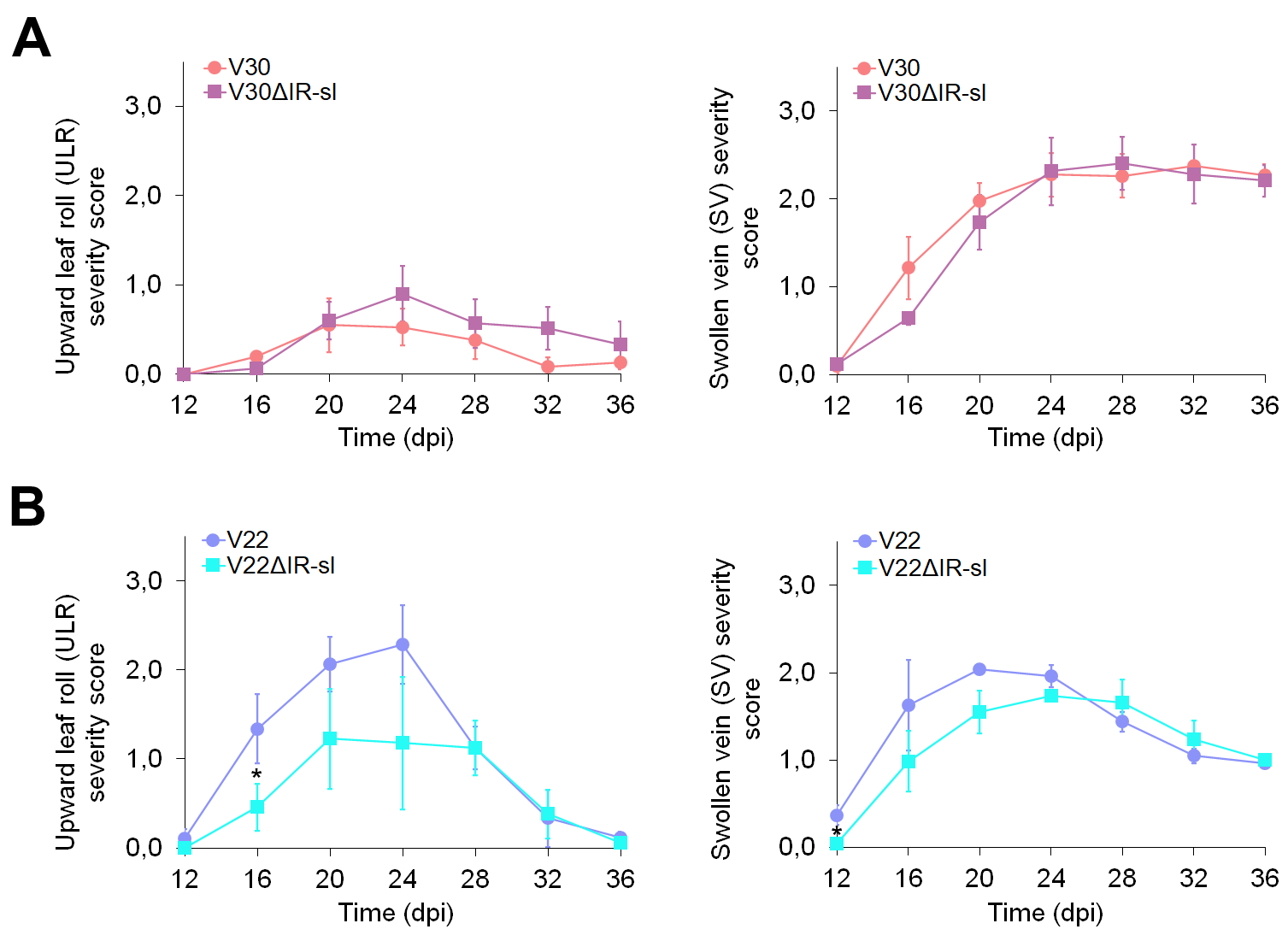

Supplement: S8 Fig — Upward leaf roll (ULR) and swollen vein (SV) severity scores obtained for plants inoculated with (A) V30, and V30ΔIR-sl and (B) V22, and V22ΔIR-sl at four-day intervals from 12 to 36 dpi. Bars represent mean ± SD. Student’s t-test levels of significance: *, p < 0.05. (TIF) [file pone.0286149.s008.tif]
