# Supplementary material for: Differences in the 3’ intergenic region and the V2 protein of two sequence variants of tomato curly stunt virus play an important role in disease pathology in Nicotiana benthamiana
Source: PLoS One. 2023 May 23;18(5):e0286149. doi: 10.1371/journal.pone.0286149 (PMC10205009; doi:10.1371/journal.pone.0286149)
Supplement: S9 Fig — (PDF) [file pone.0286149.s009.pdf]

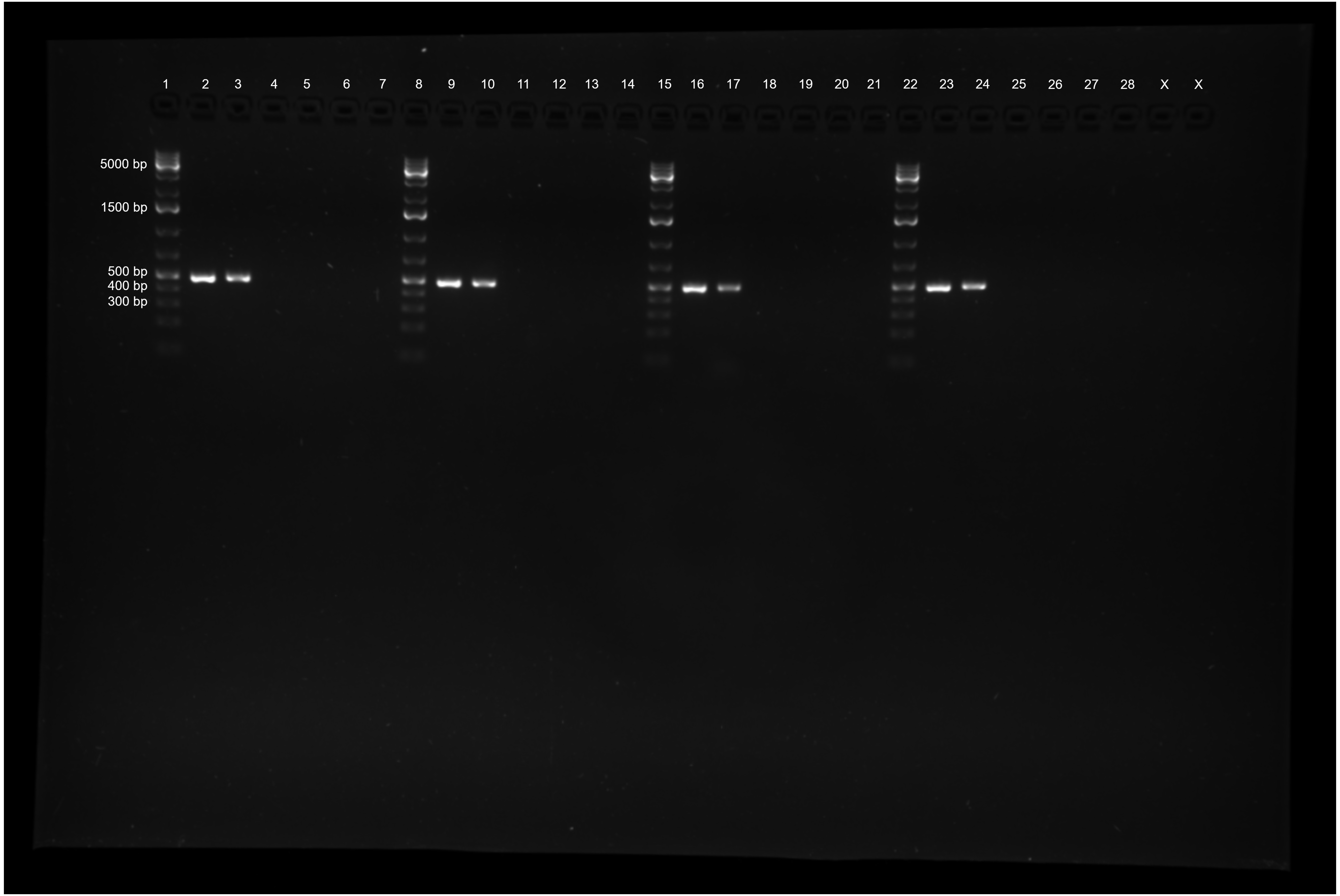

1: Molecular weight marker
2: Positive PCR control: PCR of total DNA extracted from V30-inoculated plant (C5seq_FP/RP)
3: RT-PCR of total RNA extracted from V30-inoculated plant (C5seq_**FP**/RP)
4: Negative reverse transcriptase enzyme control (V30-inoculated plant) (C5seq_**FP**/RP)
5: RT-PCR of total RNA extracted from mock-inoculated plant (C5seq_**FP**/RP)
6: Negative reverse transcriptase enzyme control (mock-inoculated plant) (C5seq_**FP**/RP)
7: PCR no template control (C5seq_FP/RP)
8: Molecular weight marker
9: Positive PCR control: PCR of total DNA extracted from V30-inoculated plant (C5seq_FP/RP)
10: RT-PCR of total RNA extracted from V30-inoculated plant (C5seq_FP/**RP**)
11: Negative reverse transcriptase enzyme control (V30-inoculated plant) (C5seq_FP/**RP**)
12: RT-PCR of total RNA extracted from mock-inoculated plant (C5seq_FP/**RP**)
13: Negative reverse transcriptase enzyme control (mock-inoculated plant) (C5seq_FP/**RP**)
14: PCR no template control (C5seq_FP/RP)
15: Molecular weight marker
16: Positive PCR control: PCR of total DNA extracted from V30-inoculated plant (V30M_FP/RP)
17: RT-PCR of total RNA extracted from V30-inoculated plant (V30M_**FP**/RP)
18: Negative reverse transcriptase enzyme control (V30-inoculated plant) (V30M_**FP**/RP)
19: RT-PCR of total RNA extracted from mock-inoculated plant (V30M_**FP**/RP)
20: Negative reverse transcriptase enzyme control (mock-inoculated plant) (V30M_**FP**/RP)
21: PCR no template control (V30M_FP/RP)
22: Molecular weight marker
23: Positive PCR control: PCR of total DNA extracted from V30-inoculated plant (V30M_FP/RP)
24: RT-PCR of total RNA extracted from V30-inoculated plant (V30M_FP/**RP**)
25: Negative reverse transcriptase enzyme control (V30-inoculated plant) (V30M_FP/**RP**)
26: RT-PCR of total RNA extracted from mock-inoculated plant (V30M_FP/**RP**)
27: Negative reverse transcriptase enzyme control (mock-inoculated plant) (V30M_FP/**RP**)
28: PCR no template control (V30M_FP/RP)

Primers used for PCR in brackets with primer used for cDNA synthesis in bold

Lanes 1-7 used for Figure 6, panel R7
Lanes 8-14 used for Figure 6, panel R1
Lanes 15-21 used for Figure 6, panel R5
Lanes 22-28 used for Figure 6, panel R3

Image captured using: ChemiDoc™ MP with UV Trans illumination of agarose gel stained with ethidium bromide (auto exposure)

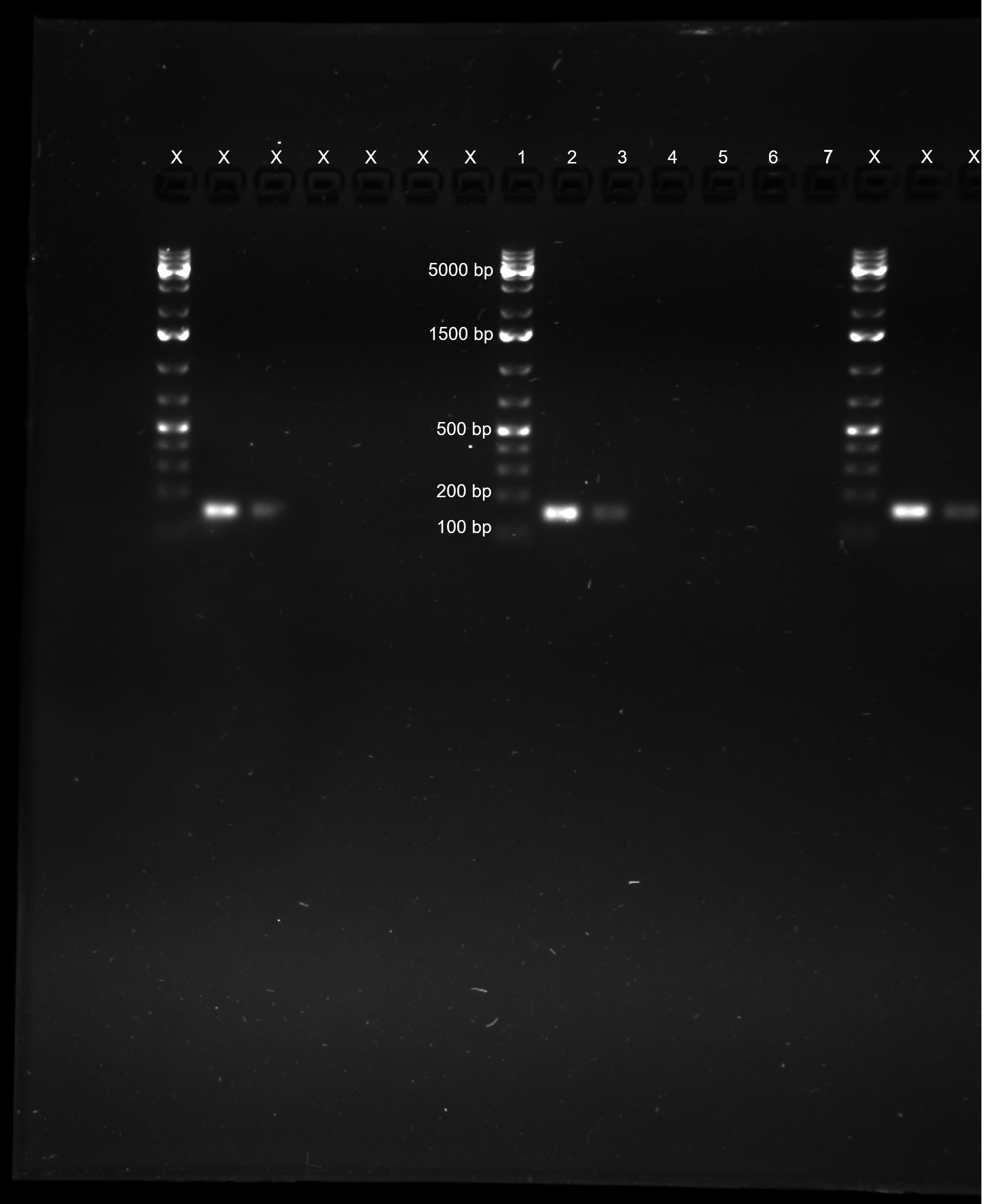

1: Molecular weight marker
2: Positive PCR control: PCR of total DNA extracted from V30-inoculated plant (V30IR_FP/RP)
3: RT-PCR of total RNA extracted from V30-inoculated plant (V30IR_FP/**RP**)
4: Negative reverse transcriptase enzyme control (V30-inoculated plant) (V30IR_FP/**RP**)
5: RT-PCR of total RNA extracted from mock-inoculated plant (V30IR_FP/**RP**)
6: Negative reverse transcriptase enzyme control (mock-inoculated plant) (V30IR_FP/**RP**)
7: PCR no template control (V30IR_FP/RP)

Primers used for PCR in brackets with primer used for cDNA synthesis in bold

Lanes 1-7 used for Figure 6, panel R4

Image captured using: ChemiDoc™ MP with UV Trans illumination of agarose gel stained with ethidium bromide (auto exposure)

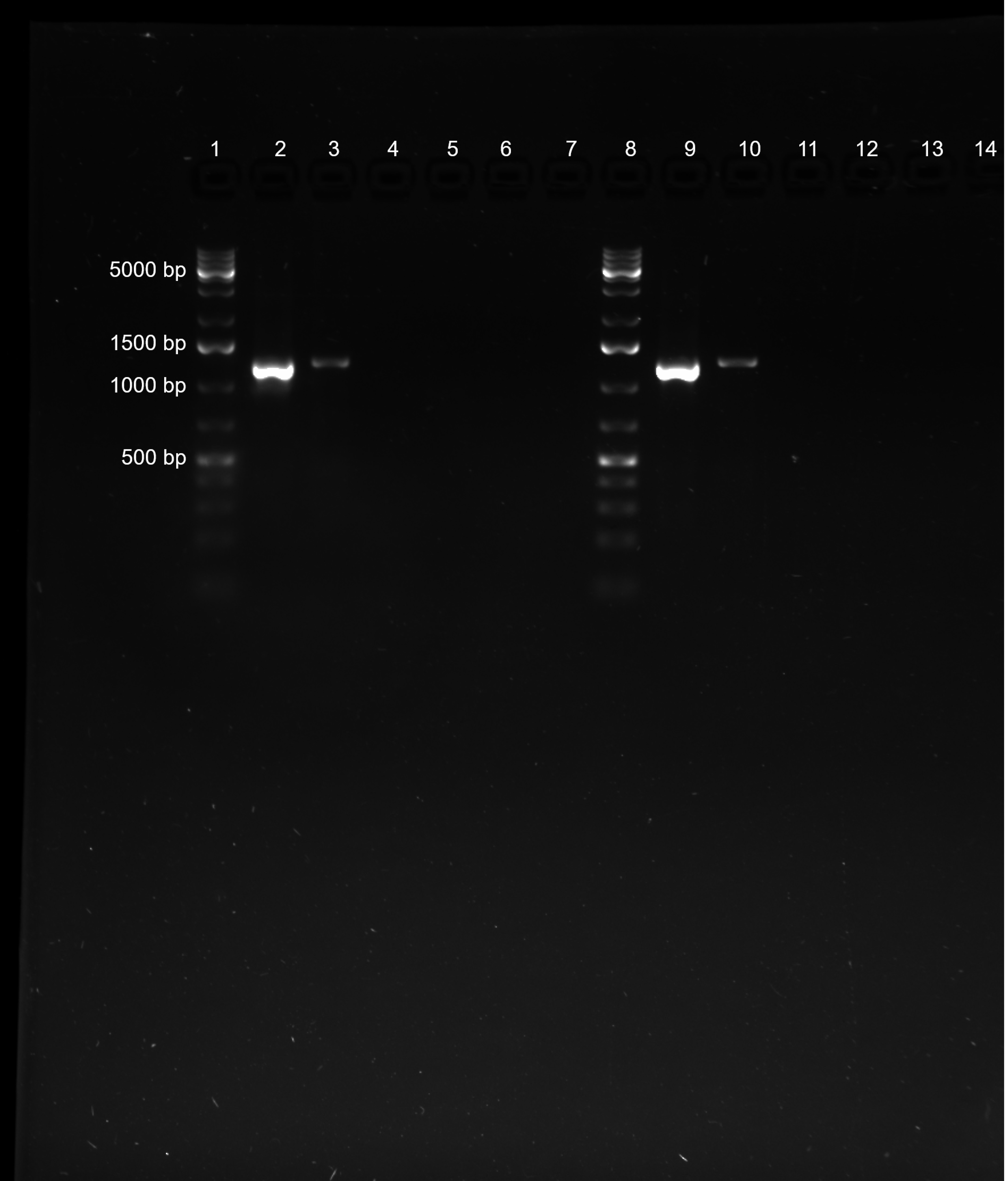

1: Molecular weight marker
2: Positive PCR control: PCR of total DNA extracted from V30-inoculated plant (C5M_FP/C1T_RP)
3: RT-PCR of total RNA extracted from V30-inoculated plant (C5M_**FP**/C1T_RP)
4: Negative reverse transcriptase enzyme control (V30-inoculated plant) (C5M_**FP**/C1T_RP)
5: RT-PCR of total RNA extracted from mock-inoculated plant (C5M_**FP**/C1T_RP)
6: Negative reverse transcriptase enzyme control (mock-inoculated plant) (C5M_**FP**/C1T_RP)
7: PCR no template control (C5M_FP/C1T_RP)
8: Molecular weight marker
9: Positive PCR control: PCR of total DNA extracted from V30-inoculated plant (C5M_FP/C1T_RP)
10: RT-PCR of total RNA extracted from V30-inoculated plant (C5M_FP/C1T_**RP**)
11: Negative reverse transcriptase enzyme control (V30-inoculated plant) (C5M_FP/C1T_**RP**)
12: RT-PCR of total RNA extracted from mock-inoculated plant (C5M_FP/C1T_**RP**)
13: Negative reverse transcriptase enzyme control (mock-inoculated plant) (C5M_FP/C1T_**RP**)
14: PCR no template control (C5M_FP/C1T_RP)

Primers used for PCR in brackets with primer used for cDNA synthesis in bold

Lanes 1-7 used for Figure 6, panel R6
Lanes 8-14 used for Figure 6, panel R2

Image captured using: ChemiDoc™ MP with UV Trans illumination of agarose gel stained with ethidium bromide (auto exposure)

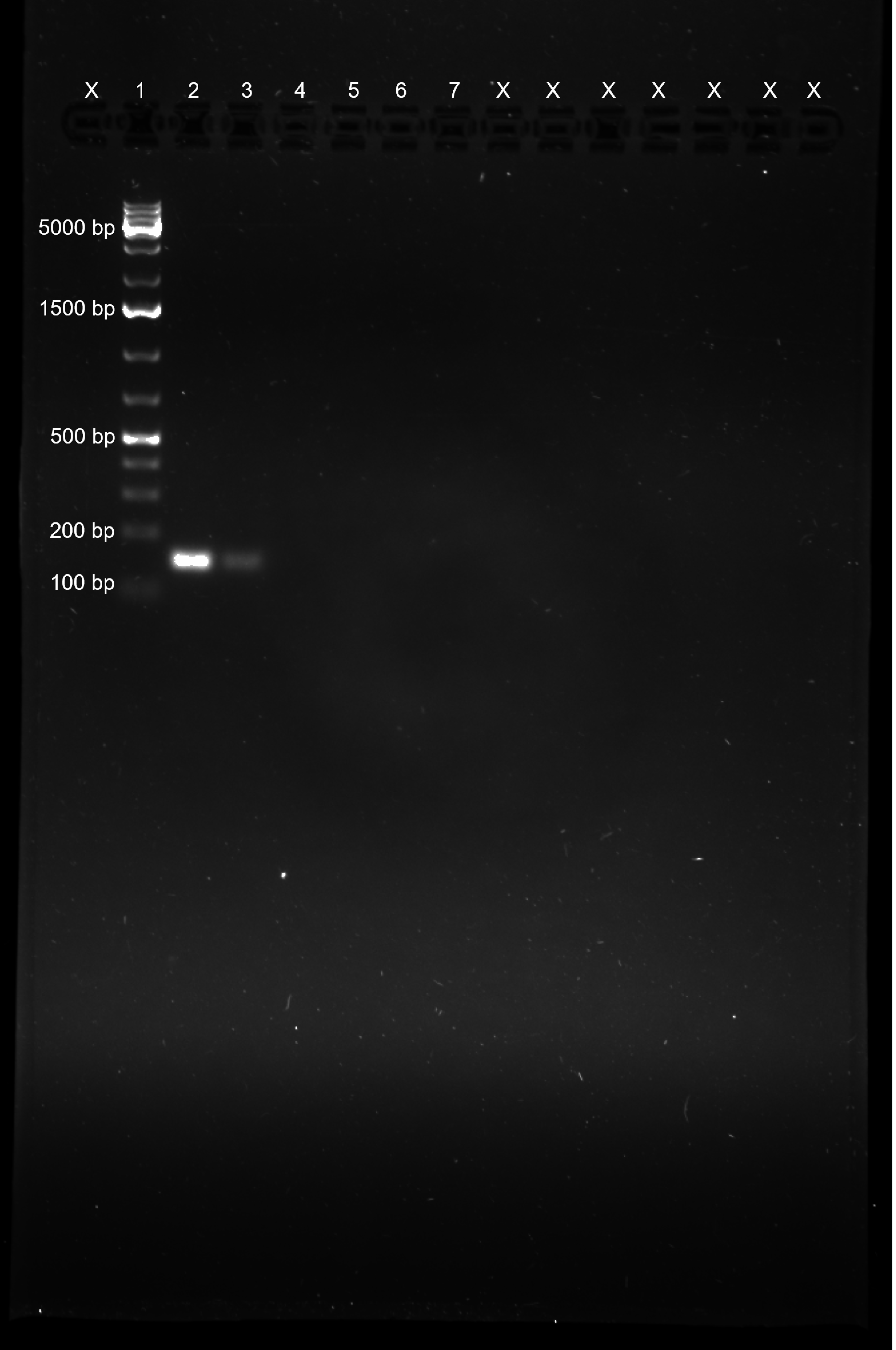

1: Molecular weight marker
2: Positive PCR control: PCR of total DNA extracted from V30-inoculated plant (V30IR_FP/RP)
3: RT-PCR of total RNA extracted from V30-inoculated plant (V30IR_**FP**/RP)
4: Negative reverse transcriptase enzyme control (V30-inoculated plant) (V30IR_**FP**/RP)
5: RT-PCR of total RNA extracted from mock-inoculated plant (V30IR_**FP**/RP)
6: Negative reverse transcriptase enzyme control (mock-inoculated plant) (V30IR_**FP**/RP)
7: PCR no template control (V30IR_FP/RP)

Primers used for PCR in brackets with primer used for cDNA synthesis in bold

Lanes 1-7 used for Figure 6, panel R8

Image captured using: ChemiDoc™ MP with UV Trans illumination of agarose gel stained with ethidium bromide (auto exposure)

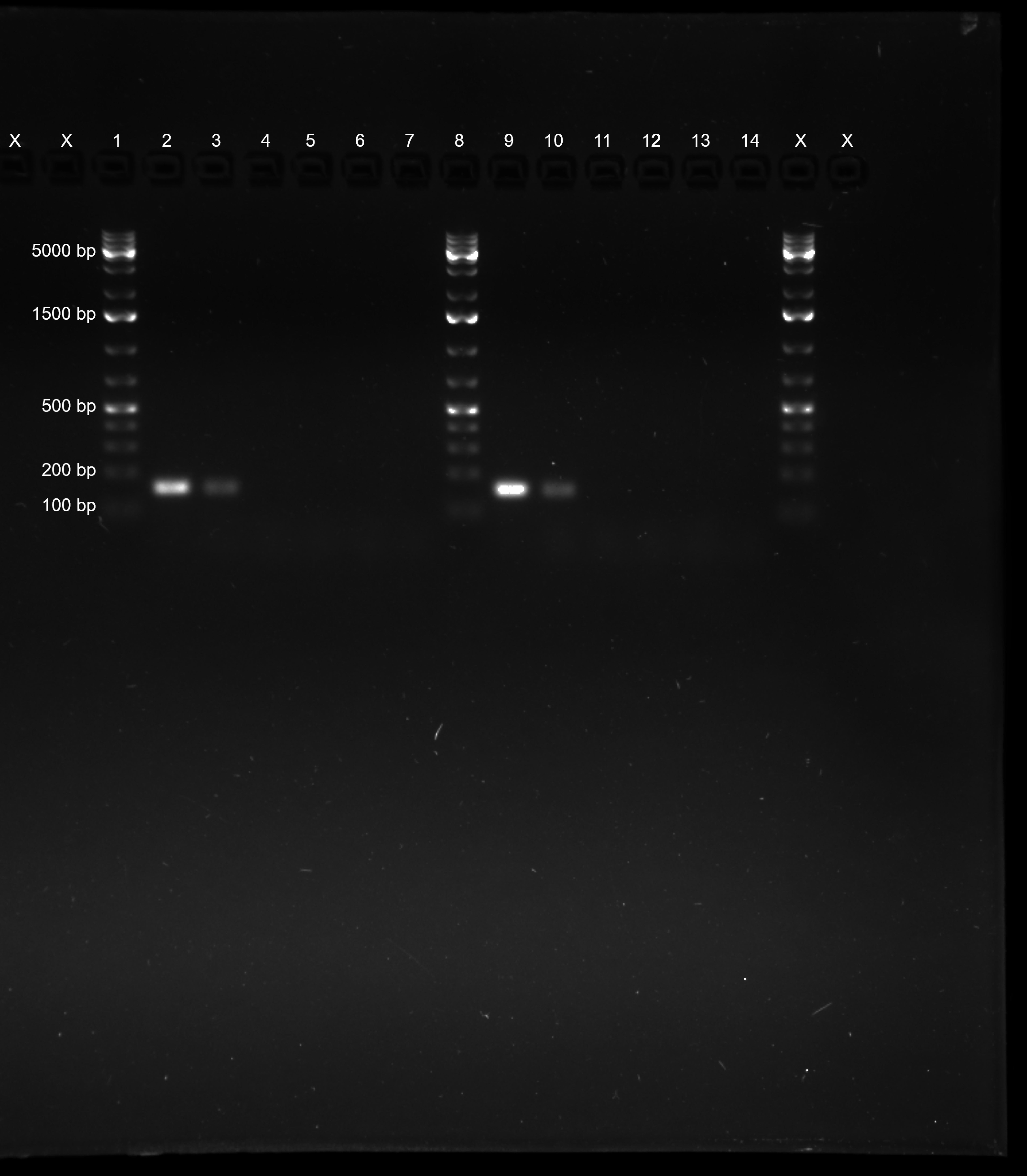

1: Molecular weight marker
2: Positive PCR control: PCR of total DNA extracted from V22-inoculated plant (V22IR_FP/RP)
3: RT-PCR of total RNA extracted from V22-inoculated plant (V22IR_**FP**/RP)
4: Negative reverse transcriptase enzyme control (V22-inoculated plant) (V22IR_**FP**/RP)
5: RT-PCR of total RNA extracted from mock-inoculated plant (V22IR_**FP**/RP)
6: Negative reverse transcriptase enzyme control (mock-inoculated plant) (V22IR_**FP**/RP)
7: PCR no template control (V22IR_FP/RP)
8: Molecular weight marker
9: Positive PCR control: PCR of total DNA extracted from V22-inoculated plant (V22IR_FP/RP)
10: RT-PCR of total RNA extracted from V22-inoculated plant (V22IR_FP/**RP**)
11: Negative reverse transcriptase enzyme control (V22-inoculated plant) (V22IR_FP/**RP**)
12: RT-PCR of total RNA extracted from mock-inoculated plant (V22IR_FP/**RP**)
13: Negative reverse transcriptase enzyme control (mock-inoculated plant) (V22IR_FP/**RP**)
14: PCR no template control (V22IR_FP/RP)

Primers used for PCR in brackets with primer used for cDNA synthesis in bold

Lanes 1-7 used for Supplementary Figure 6, panel R10
Lanes 8-14 used for Supplementary Figure 6, panel R9

Image captured using: ChemiDoc™ MP with UV Trans illumination of agarose gel stained with ethidium bromide (auto exposure)
