# Supplementary material for: Differences in the 3’ intergenic region and the V2 protein of two sequence variants of tomato curly stunt virus play an important role in disease pathology in Nicotiana benthamiana
Source: PLoS One. 2023 May 23;18(5):e0286149. doi: 10.1371/journal.pone.0286149 (PMC10205009; doi:10.1371/journal.pone.0286149)
Supplement: S1 Table — (DOCX) [file pone.0286149.s010.docx]

**S1 Table. Description of sequence changes made to generate ToCSV variant IR and V2 sequence swap mutants.**

| **Mutant** | **Template** | **Sequence changes** |
| --- | --- | --- |
| V30ΔIR-s | V30 | V30 nt 61-138 replaced with V22 nt 61-137 (partial IR 3’ region) |
| V30ΔIR-sr | V30 | V30 nt 108-138 replaced with V22 nt 108-137 (partial IR 3’ region to right of TATA box) |
| V22ΔIR-s | V22 | V22 nt 61-137 replaced with V30 nt 61-138 (partial IR 3’ region) |
| V22ΔIR-sr | V22 | V22 nt 108-137 replaced with V30 nt 108-138 (partial IR 3’ region to right of TATA box) |
| V30ΔV2-s | V30 | V30 nt 139-489 replaced with V22 nt 138-488 (full canonical V2 ORF) |
| V22ΔV2-s | V22 | V22 nt 138-488 replaced with V30 nt 139-489 (full canonical V2 ORF) |
