# Supplementary material for: Differences in the 3’ intergenic region and the V2 protein of two sequence variants of tomato curly stunt virus play an important role in disease pathology in Nicotiana benthamiana
Source: PLoS One. 2023 May 23;18(5):e0286149. doi: 10.1371/journal.pone.0286149 (PMC10205009; doi:10.1371/journal.pone.0286149)
Supplement: S2 Table — (DOCX) [file pone.0286149.s011.docx]

**S2 Table. Primers used in this study.**

| **Primer** | **Sequence (5’-3’)** | **Purpose** |
| --- | --- | --- |
| ToCSVseq_FP | GTCGTGCTCCACCATGTTG | PCR, sequencing (IR) |
| ToCSVseq_RP | CACCACGAACCTTTTACCCG | PCR, sequencing (IR) |
| V22IR_FP2 | AAACTCCTAAAGCGGCCATC | PCR, sequencing (IR) |
| V22V2_FP | GTGCGGATGTCATAATGTGG | PCR, sequencing (V2) |
| V22V2_RP | GACCAGGACCCTTTGTAATGTC | PCR, sequencing (V2) |
| ΔV2V27S_FP | CTTGCAatcgATTGAGTCCACTTATGAGCC | Mutagenesis to generate V22ΔV2-V27S |
| ΔV2V27S_RP | GGACTCAATcgatTGCAAGTATTTTATAGCAA GC |  |
| ΔV2T58S_FP | GAAGCGtCCCGCCGATATAATCATTTCCAC | Mutagenesis to generate V22ΔV2-T58S |
| ΔV2T58S_RP | GATTATATCGGCGGGaCGCTTCGACATAAT C |  |
| C5seq_FP | TGGGATCCACTGTTAAACGA | cDNA synthesis, PCR (for RT-PCR) |
| C5seq_RP | TCACCACGAACCTTTTACCC | cDNA synthesis, PCR (for RT-PCR) |
| GAPDH_FP | CTGTTATTGGAGGAGGGAACAA | qPCR |
| GAPDH_RP | AGTCTTTCCTACCATGCCAAC | qPCR |
| V2_FP | CGACAGCCCGTTCACCAG | qPCR |
| V2_RP | GCCTGTACGTCCATGATCGTC | qPCR |
| V30IR_FP | GCATTTCAAAATTCAAACTCCTAAAGC | cDNA synthesis, PCR (for RT-PCR) |
| V30IR_RP | AGCAGATAACTAAGCGATAAGGC | cDNA synthesis, PCR (for RT-PCR) |
| V22IR_FP | GTAATTTGTGGCAAAGTAATTGGAATTTC | cDNA synthesis, PCR (for RT-PCR) |
| V22IR_RP | GCTTTAACGCCTCAATTTCATTGG | cDNA synthesis, PCR (for RT-PCR) |
| V30M_FP | GAAACGACCAGTCTGAGGCT | cDNA synthesis, PCR (for RT-PCR) |
| V30M_RP | TAAAGGCGGCATTCCCACTA | cDNA synthesis, PCR (for RT-PCR) |
| C5M_FP | ACGATCATGGACGTACAGGC | cDNA synthesis, PCR (for RT-PCR) |
| C1T_RP | CCCTCAACCAGCCACTGTAC | cDNA synthesis, PCR (for RT-PCR) |

lower case nucleotide = target mutation/s.
