# Supplementary material for: Differences in the 3’ intergenic region and the V2 protein of two sequence variants of tomato curly stunt virus play an important role in disease pathology in Nicotiana benthamiana
Source: PLoS One. 2023 May 23;18(5):e0286149. doi: 10.1371/journal.pone.0286149 (PMC10205009; doi:10.1371/journal.pone.0286149)
Supplement: S3 Table — (DOCX) [file pone.0286149.s012.docx]

**S3 Table. Monopartite begomovirus isolates used in this study.**

| **Begomovirus species** | **Abbreviation** | **Isolate** | **Accession number** | **Pairwise sequence identity (%)** | |
| --- | --- | --- | --- | --- | --- |
|  |  |  |  | **ToCSV-[ZA:Mks30:08]** | **ToCSV-[ZA:Mks22:07]** |
| *Pepper yellow vein Mali virus* | PepYVMLV | Mali | AY502935 | 82.3 | 82.1 |
| *Tobacco leaf curl Comoros virus* | TbLCKMV | Comoros/Simboussa/2004 | AM701760 | 78.1 | 79.0 |
| *Tobacco leaf curl Zimbabwe virus* | TbLCZV | Zimbabwe | AF350330 | 83.8 | 84.5 |
| *Tomato curly stunt virus* | ToCSV | South Africa/Onderberg/1998 | AF261885 | 99.0 | 95.5 |
| *Tomato leaf curl Anjouan virus* | ToLCAnV | Comoros/Ouani/2004 | AM701758 | 78.0 | 77.9 |
| *Tomato leaf curl Arusha virus* | ToLCArV | Tanzania/Kilimandjaro/2005 | EF194760 | 79.7 | 80.4 |
| *Tomato leaf curl Burkina Faso virus* | ToLCBFV | Burkina Faso-Loumbila-Tomate51B1-2013 | KX853168 | 79.5 | 79.5 |
| *Tomato leaf curl Comoros virus* | ToLCKMV | Mayotte/Kahani/2003 | AJ865340 | 79.8 | 80.9 |
| *Tomato leaf curl Diana virus* | ToLCDiV | Madagascar/Namakely/2001 | AM701765 | 79.3 | 79.6 |
| *Tomato leaf curl Ghana virus* | ToLCGV | Ghana/Akumadan/2006 | EU350585 | 78.5 | 78.3 |
| *Tomato leaf curl Kunene virus* | ToLCKunV | NA-2019 | MT045996 | 78.9 | 79.0 |
| *Tomato leaf curl Madagascar virus* | ToLCMGV | Madagascar/Morondova/2001/Menabe | AJ865338 | 79.9 | 80.7 |
| *Tomato leaf curl Mali virus* | ToLCMLV | Mali | AY502936 | 78.8 | 79.0 |
| *Tomato leaf curl Namakely virus* | ToLCNaV | Madagascar/Namakely/2001 | AM701764 | 79.9 | 80.4 |
| *Tomato leaf curl Nigeria virus* | ToLCNGV | Nigeria/2006 | FJ685621 | 79.8 | 79.8 |
| *Tomato leaf curl Seychelles virus* | ToLCSCV | Seychelles/Val d'Endor/2004 | AM491778 | 78.5 | 78.9 |
| *Tomato leaf curl Tanzania virus* | ToLCTZV | TZ-Ten-05 | DQ519575 | 78.8 | 79.6 |
| *Tomato leaf curl Toliara virus* | ToLCToV | Madagascar/Miandrivazo/2001 | AM701768 | 79.2 | 79.6 |
| *Tomato leaf curl Uganda virus* | ToLCUV | Uganda/Iganga/2005 | DQ127170 | 79.3 | 80.3 |
| *Tomato yellow leaf curl Mali virus* | TYLCMLV | Burkina Faso/Tom141/2013 | LM651400 | 79.0 | 79.3 |
