# Supplementary material for: Differences in the 3’ intergenic region and the V2 protein of two sequence variants of tomato curly stunt virus play an important role in disease pathology in Nicotiana benthamiana
Source: PLoS One. 2023 May 23;18(5):e0286149. doi: 10.1371/journal.pone.0286149 (PMC10205009; doi:10.1371/journal.pone.0286149)
Supplement: S4 Table — (DOCX) [file pone.0286149.s013.docx]

**S4 Table. Upward leaf roll (ULR) symptom severity scoring system for *Nicotiana benthamiana*.**

| **Score** | **Description** |
| --- | --- |
| 0 | Leaf edges normal |
| 1 | Leaf edges turned up 1 to 7 zones, or mild ULR 1 to 4 zones |
| 2 | Leaf edges turned up 2 to 7 zones and mild/narrow ULR ≤4 zones, or narrow/mild ULR 5 to 7 zones, or broad/severe ULR 1 to 4 zones |
| 3 | Severe ULR ≥ 5 zones, or entire leaf edge rolled upward and onto itself |
