# Supplementary material for: Differences in the 3’ intergenic region and the V2 protein of two sequence variants of tomato curly stunt virus play an important role in disease pathology in Nicotiana benthamiana
Source: PLoS One. 2023 May 23;18(5):e0286149. doi: 10.1371/journal.pone.0286149 (PMC10205009; doi:10.1371/journal.pone.0286149)
Supplement: S5 Table — (DOCX) [file pone.0286149.s014.docx]

**S5 Table. Swollen leaf vein (SV) symptom severity scoring system for *Nicotiana benthamiana*.**

| **Score** | **Description** |
| --- | --- |
| 0 | Leaf veins normal |
| 1 | Slight SV to mild SV over entire leaf, or moderate SV over less than half of leaf |
| 2 | Moderate SV more than half of leaf only, or moderate SV more than half of leaf with some severe SV, or severe SV less than half of leaf |
|  |  |
| 3 | Severe SV more than half of leaf, or dense SV over entire leaf |
