# Supplementary material for: Differences in the 3’ intergenic region and the V2 protein of two sequence variants of tomato curly stunt virus play an important role in disease pathology in Nicotiana benthamiana
Source: PLoS One. 2023 May 23;18(5):e0286149. doi: 10.1371/journal.pone.0286149 (PMC10205009; doi:10.1371/journal.pone.0286149)
Supplement: S6 Table — (DOCX) [file pone.0286149.s015.docx]

**S6 Table. Predicted ORFs for ToCSV V30 and V22 genomes encoding proteins ≥60 aa in length.**

| **ORF** | **Start codon nt position (V30/V22)** | **Stop codon nt position**  **(V30/V22)** | **Protein length (aa) (V30/V22)** |
| --- | --- | --- | --- |
| V1 | 299/298 | 1075/1074 | 258/258 |
| V2 | 139/138 | 480/488 | 113/116 |
| C1 | 2612/2611 | 1533/1532 | 359/359 |
| C2 | 1624/1623 | 1217/1216 | 135/135 |
| C3 | 1476/1475 | 1072/1071 | 134/134 |
| C4 | 2455/2454 | 2198/2197 | 85/85 |
| C5* | 610/609 | 428/427 | 60/60 |
| C6* | 585/584 | 154/258 | 143/108 |

* = putative ORF.
