# Supplementary material for: Differences in the 3’ intergenic region and the V2 protein of two sequence variants of tomato curly stunt virus play an important role in disease pathology in Nicotiana benthamiana
Source: PLoS One. 2023 May 23;18(5):e0286149. doi: 10.1371/journal.pone.0286149 (PMC10205009; doi:10.1371/journal.pone.0286149)
Supplement: S7 Table — (DOCX) [file pone.0286149.s016.docx]

**S7 Table. Predicted viral protein changes for V30ΔV2-s and V22ΔV2-s mutants following standard code.**

| **Protein** | **Changes in V30ΔV2-s from V30** | **Changes in V22ΔV2-s from V22** |
| --- | --- | --- |
| CP (V1) | Ala36🡪Val; Lys55🡪Arg | Val36🡪Ala; Arg55🡪Lys |
| V2 | Entire V2 swapped, protein identical to V22 V2 | Entire V2 swapped, protein identical to V30 V2 |
| C5* | Ile45🡪Val; Leu50🡪Pro | Val45🡪Ile; Pro50🡪Leu |
| C6* | Partial C6 swapped, protein identical to V22 C6. | Partial C6 swapped, protein identical to V30 C6. |

* = putative protein, CP = coat protein.
